# Spatial distribution, sources and health risk assessment of heavy metals in topsoil around oil and natural gas drilling sites, Andhra Pradesh, India

**DOI:** 10.1038/s41598-023-36580-9

**Published:** 2023-06-30

**Authors:** Keshav Krishna Aradhi, Babu Mallesh Dasari, Dasaram Banothu, Satyanarayanan Manavalan

**Affiliations:** 1 CSIR-National Geophysical Research Institute (Council of Scientific and Industrial Research), Habsiguda, Hyderabad, 500007 India; 2 Academy of Scientific and Innovative Research (AcSIR), Ghaziabad, 201002 India

**Keywords:** Environmental sciences, Risk factors

## Abstract

Soils are usually the interface between human activity and environmental components that must be conserved and protected. As a result of rising industrialization and urbanization, activities such as exploration and extraction operations lead to the release of heavy metals into the environment. This study presents distribution of six heavy metals (As, Cr, Cu, Ni, Pb and Zn) in 139 top soil samples collected in and around oil and natural gas drilling sites at a sampling density of 1 site/12 km^2^. The results indicated the concentration ranged from 0.1 to 16 mg/kg for As, 3–707 mg/kg for Cr, 7–2324 mg/kg for Cu, 14–234 mg/kg for Ni, 9–1664 mg/kg for Pb, and 60–962 mg/kg for Zn. The contamination of soil was estimated on the basis of Index of geo accumulation (I_geo_), enrichment factor (E_f_), and contamination factor (C_f_). Further, spatial distribution pattern maps indicated that the pollution levels for Cu, Cr, Zn, and Ni were higher around drilling sites of the study area relative to other regions. Using exposure factors for the local population and references from the USEPA’s integrated database, potential ecological risk indices (PERI) and health risk assessments were made. The hazard index (HI) values of Pb (in adults) and Cr, Pb (in children) exceeded the recommended limit of HI = 1, indicating the non-carcinogenic risks. Total carcinogenic risk (TCR) calculations revealed Cr (in adults) and As, Cr (in children) levels in soils exceeded the threshold value of 1.0E − 04, indicating significant carcinogenic risk due to high metal concentrations in the study area. These results may assist in determining the soil’s present state and its effect due to extraction strategies used during drilling process and initiate few remedial techniques, particularly for proper management strategies in farming activities to decrease point and non-point source of contamination.

## Introduction

Soil contamination by heavy metals has been a major problem in recent years. These elements can accumulate in plants and animals, ultimately entering the human food chain. The quality of water, soil, and aquatic systems is strongly influenced by the industrial sector. As the sector expands, inefficient waste management has become a significant obstacle to overcome. Soil samples are an effective way to screen heavy metal contamination as anthropogenic heavy metals are typically placed in top soils^1,2^. Extraction effluents contain a variety of pollutants like inorganic contaminants including heavy metals, which have been linked to serious health and environmental issues. Heavy metals are the most important soil pollutants originating from geogenic and anthropogenic sources because they are persistently harmful and introduce additional health hazards which must be regulated in our environment. These toxins may be spread to other sections of the environment in a way that is influenced by the air, the activity of transportation, and gravitation, all of which may have an effect^3–5^. However, some heavy metals are associated with soils, which are considered to serve as the main repository when introduced into the environment. Further, heavy metal contamination in soil is made worse by the fact that chemical elements and compounds can move through geochemical processes like dissolution and adsorption into the sediments below the soil layer and into the groundwater environment^6–10^.

Multiple sources, such as industrial waste, petrochemical spills which include drilling activities for oil and gas exploration, atmospheric deposition, and mine tailings, contribute to the accumulation of heavy metals^11^. Geogenic sources especially those sedimentary rocks and alluviums formed from fluvial deposits also contribute to the heavy metal budget as they act as sink for inorganic and organic metals/metalloids during the geological time scale. More emphatically, water and solid wastes generated out of oil–gas production and fracking operations are the most probable to be polluted with these heavy metals, according to an investigation^12^. Furthermore, the agriculture fields wherein the drilling activities are taken up would become unfertile for future cultivation. Farming soil contaminated with heavy metals may consequently provide major threats to the food supply and ecological responsibility related to the direct interaction of vegetation with soil. Despite the possibility that these localities face major environmental problems, there has not been a correspondingly detailed investigation to examine the level of heavy metal contamination. The scarcity of natural resources on a global scale presents a challenge for effectively segregating industrial and agricultural activities within the area. This problem is brought on by the increased difficulty in controlling the different pollutants that build up in the soil and the greater threat to agricultural production deterioration brought on by exposure to other sources of contaminants. Extremely essential is a comprehensive evaluation of the properties of urban soils^13,14^. Using appropriate indices, soil contamination can be measured^15–18^. The assessment of the quality of the soil may be carried out by using a variety of techniques, such as the calculation of the contamination factor (CF), the enrichment factor (EF), and the geo accumulation index (I_geo_). An ecological risk assessment may further examine soil quality^19–23^.

Due to the harmfulness and persistence of heavy metals in the environment, a study on their environmental geochemistry has considerably developed over the last few decades^24,25^. Recently, numerous sectors have employed the analysis of principal components to assess soil, including heavy metal contamination. This form of comparison exposes heavy metal correlations in several spatial dimensions and evaluates each metals relative importance. If the elements are more than what was prescribed or if changes were made to the natural environment, then the soil is considered to be contaminated^26,27^. Various researches have been conducted in the past few years on heavy metals pollution, source area, a pattern of dispersion, amount of pollution, and related concerns to human health in various regions around the world^28–30^. The activities of the oil sector have caused ecological disruption and decreased diversity in these areas, harming the environment as a whole^31^. The published literature states that numerous contemporary research on soil pollution have been carried out to investigate harmful compounds, especially in industrialized nations. Metals in surface soils pose serious risks to human health through a variety of exposure pathways, including ingestion, inhalation, and skin contact^32–37^. A global issue of growing concern is the buildup of heavy metals in urban soils and surfaces^38^^.^ According to areas close to industrial activity suffer greatly from contaminated air, soil, and water^39,40^. Studies on the levels of metal concentrations, spatial distribution of elements, and potential risks related to a heavy metal concentration in the soil near oil refineries have been carried out in India^41^, Iran^42,43^, and Nepal^44^. The present study was carried out keeping in view the growing demand for non-renewable energy sources using advanced technologies, especially the un-conventional techniques using hydraulic fracturing which have been predicted to damage the ecology in near future by so-called back water or produced water containing different chemicals including inorganic and organic compounds known to be detrimental to human health. The originality in the present work is the generation of new data in the study area and would fill the gap by providing baseline information regarding heavy metal distribution and accumulation in the soil, thereby to sustain and protect the environment, with objectives (i) to evaluate the distribution of arsenic (As), chromium (Cr), copper (Cu), lead (Pb), nickel (Ni) and zinc (Zn) in soil around oil and natural gas drilling sites using multivariate statistical tools (principal component analysis), (ii) soil pollution indicators were also evaluated to diagnose the level of contamination like, geoaccumulation index (I_geo_), enrichment factor (EF), contamination factor (CF), and potential ecological risk index (PERI) (iii) Health risk classified in relation to two adverse health effects of chemical elements on humans, non-carcinogenic and carcinogenic effects were assessed using HQ and HI from so called risk assessment methodology^44,45^. Further, these studies will also be beneficial in identifying the major contaminants and help in combating and designing an approach to combat the discharge and release of toxins in the study atmosphere.

## Materials and methods

### Study area

**Figure 1:**
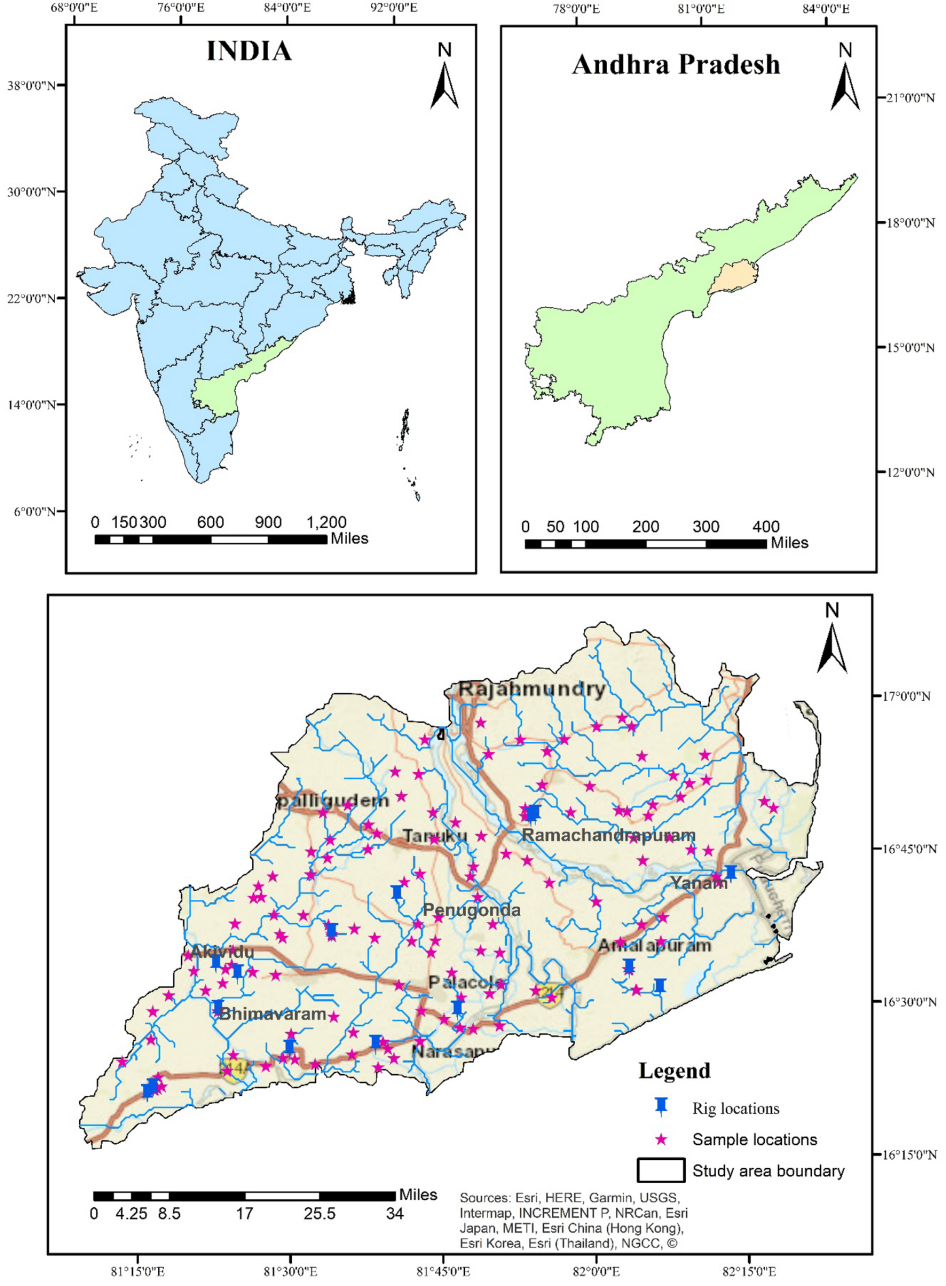
Sample location map of the study area (maps were generated with software ArcGIS 10.7 http://www.esri.com).

The present study was carried out to understand soil contamination around the oil and gas drilling sites in East & West Godavari districts, Andhra Pradesh (Fig. 1). The figure shows the spread of different oil and gas drilling plants situated in the study area namely, Konalapalli, Thallakodu, Prathallamaraka, Nagendrapuram, Arjunudupalem, Sitharamapuram, Elamanchili, Mandapeta, Kalvacherla, Yedurulanka, Challapalli, Bantumilli, Malleshwaram, and Mogallu.The study area's climate can be described as a tropical monsoon climate with distinct seasons. The temperature conditions during the different seasons like summer, rainy, and winter, showing average temperatures ranging from 30 to 40 °C, 25 to 35 °C, and 20 to 30 °C, respectively.

### Geology of the study area

**Figure 2:**
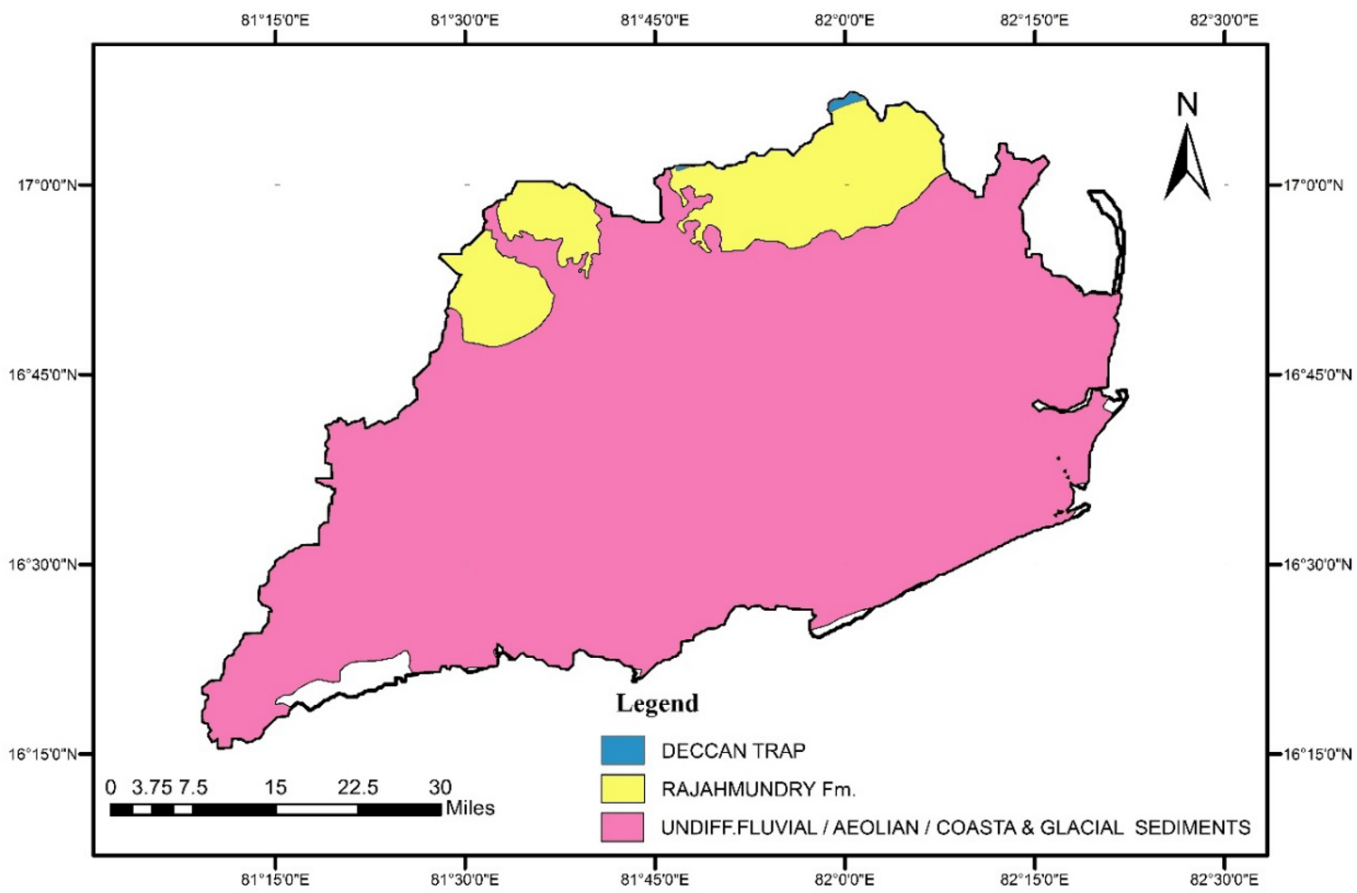
Geology map of the study area (maps were generated with software ArcGIS 10.7 http://www.esri.com).

The soil type in the districts are alluvial clay, sandy clay, and black clay. The Geology of the study area (Fig. 2) basement is Archaean crystallines overlined by Gondwana (Tirupathi) sandstones, Deccan Traps, Rajahmundry sandstone formations, and Alluvial sediments^46^. Mineralogical information reveals that the pyroxenes, amphiboles and magnetite were dominated compared to ilmenite, rutile, leucoxene, zircon, monazite and few quantities of sillimanite and garnet lesser quantities of sillimanite and garnet representing their origin from more mafic rocks^47^. The study area in East and West Godavari region preserves a geological record from Proterozoic to recent representing Eastern Ghat Mobile Belt (EGMB) to Quaternary Alluvium. The NNE-SSW to NE-SW trending EGMB is surrounded by Singhbhum Craton in the South and partly by Bastar Craton in the east. The area is significant due to the presence of the Precambrian Mobile Belt of Peninsular India consisting of deformed crustal segments and the thick repository of Meso- to Neoproterozoic Gondwana sedimentary sequences formed in the Godavari Graben. The sedimentary rocks show a NW–SE trend and is found to lie between the Dharwar Craton and the Bastar Craton almost perpendicular to EGMB. The rifting and associated tectonics during Gondwana from Permian to Carboniferous resulted in the deposition of thick sedimentary packages representing divergent sedimentary environments from Glacio-lacustrine to arid and fluvial environments and depositional history which display multiple sets of faults signifying considerably long deformational history. The sedimentary package is represented by sandstone and shale of Gondwana while the tertiaries consists of alluviums including black clay with sand, black/brown silty clay etc. The sedimentary package and the alluviums act as sink for organic and inorganic metal/metalloids in these fluvial deposits contributed to the heavy metal budget in the region forming one of the prominent geogenic source^48^.

### Sampling and preparation

A total of 139 soil samples were collected from the entire study area at a sampling density of 1 sample/12 km^2^ grid. Soil sampling was done with a shovel from the surface, at a depth of 20 cm, to investigate the anthropogenic source of pollutants, as industrial pollutants often pollute the top layer of the soil (0–40 cm). Figure 1 shows a study area map with soil sample locations. After every sample collection the shovel was washed with Milli-Q water to avoid contamination. Soil samples were stored in self-locking polythene bags and dried in the open sun and finely powdered using an agate mortar to a 250-mesh size (US Standard) for chemical analysis. The global positioning system was used to record the geographic location of each sampling location.

Finely powdered representative samples were chemically dissolved in supra pure HF and HNO_3_ (7:3 ratio) in HDPE vials (Savillex®, USA) taking 0.05 g and completing the digestion by heating the content at 70 °C for about 48 h.  Rh^49^ was used as an internal standard before making the final volume to 250 ml. The duplicates and blank were also prepared following the same procedure. Clear solutions were obtained for all the samples which were analyzed for trace elements (As, Cr, Cu, Ni, Pb and Zn) and rare earth elements (La to Lu) using a high resolution inductively coupled plasma mass spectrometry (HR-ICP-MS) (Make: Nu Instruments, UK; Model: ATTOM®) at CSIR-National Geophysical Research Institute, Hyderabad.

### Quality assurance and quality control

Geochemical certified reference materials SO-1, SO-2, SO-3, SO-4 from CANMET, Canada, and JSd-1, JSd-2, JSd-3 from GSJ, Japan were utilized for calibration and checking accuracy/precision of the analysis. The analysis was carried out at moderate resolution (R = 300) with peak scan mode by which the analytes of interest is precisely determined sequentially^50^. In most of the cases, the precision and accuracy were found to me within acceptable limits varying from 2 to 5% RSD (Table 1).

**Table 1:** Analytical results of the soil standard reference materials SO-1 and SO-2 in comparison with the certified reference values.

CRM	As	Cr	Cu	Ni	Pb	Zn
SO-1	2.0 (2.0)	178.5 (170.0)	57.2 (61.0)	90.3 (92.0)	19.5 (20.0)	141.9 (144.0)
SO-2	1.9 (1.1)	17.5 (12.3)	7.1 (8.0)	7.8 (8.0)	21.4 (20.0)	117.0 (115.0)

First row indicates measured value

Second row in brackets indicate certified values.

### Geographical information system

A Geographical information system (GIS) facilitates the collection and modification of a vast array of geographical data and presents the spatial distribution of all parameters^51^. Geographic information systems (GIS) enable the modification of all parameters affecting soil quality. This provides individuals and policymakers with a comprehensive understanding of the situation. Using the inverse distance-weighted interpolation method of the ArcGIS-10.7 program (CSIR-NGRI, Hyderabad, India), a map of the geographic distribution of all parameters was made.

## Analysis methods

### Multivariate statistical methods

The analytical data were processed using certain statistical techniques to examine the distribution and correlation of the parameters under study. Statistical SPSS 10.0 was used to compute and evaluate the heavy metal data using multivariate statistics like Principal component analysis (PCA)^52,53^. Principal components provide information on the most meaningful parameters which describe whole data set affording data reduction with minimum loss of original information. PCA is a powerful technique for pattern recognition that attempts to explain the variance of a large set of inter-correlated variables and transforming into a smaller set of independent (uncorrelated) variables (principal components). These components further reduce the contribution of less significant variables obtained from PCA and the new group of variables known as varifactors are extracted through rotating the axis. On the dataset, PCA was achieved using varimax normalized rotation.

### Pollution indices

For measuring heavy metal pollution in soil, a variety of single- and Integrated Indices approaches are available. These factors frequently make sure to give a qualification of pollution as opposed to quantification of contamination as a result of the many different factors that might influence their utility. The most essential difficulty is that until historical data is available, it is sometimes quite difficult to determine what the actual composition of the soil/sediment was in terms of the components of relevance. Also, the issue is the demand for an analog of non-contaminated soil/sediment if there is no historical data. This necessitates taking a sample from somewhere other than the polluted location. This poses the question of how to account for heavy metal inputs from sedimentary and lithogenic sources. Calculating environmental quality indices (soil/sediment quality indices) is the basis of quantitative techniques. These techniques are regarded as an excellent tool for modifying environmental data into information that non-specialists can understand^54^. Soil Quality Classification for Pollution Indices is shown in Table 2.

**Table 2:** Soil Quality Classification with threshold values for Pollution Indices.

Class	Qualification	I_geo_	EF	CF
0	Unpolluted	I_geo_ < 1	EF < 2	CF < 1
1	Slightly polluted	1 < I_geo_ < 2		
2	Moderately polluted	2 < I_geo_ < 3	2 < EF < 5	1 < CF < 3
3	From moderately polluted to strongly polluted	3 < I_geo_ < 4		
4	Strongly polluted	4 < I_geo_ < 5	5 < EF < 20	3 < CF < 6
5	From strongly polluted to extremely polluted		20 < EF < 40	CF > 6
6	Extremely polluted	I_geo_ > 5	EF > 40	

I_geo_, geo accumulation index; EF, enrichment factor; CF, contamination factor ( Adapted from^55,56,62^ ).

#### Index of geoaccumulation (I_geo_)

The Index of Geoaccumulation offers an evaluation of pollution by assessing modern and pre-industrial concentrations in bottom sediments. Additionally, this can be utilized in the evaluation of soil pollution^55^. The following equation was used to calculate it:

1 $$I_{geo} = {\text{ Log }}\left( {{\text{Cn}}/{1}.{\text{5 B}}_{{\text{n}}} } \right)$$

where Cn is the element's observed concentration in the samples, and Bn is either from the literature or directly measured in texturally similar uncontaminated soils. The constant 1.5 allows us to investigate regular variabilities in the concentration of a specific material and minor human impacts in the environment. In this investigation, we used a modified calculation based on Loska Equation^56^, where Cn signified the content of a certain element in the soil analyzed, and Bn represented the content of elements in the earth's crust^57^.

#### Enrichment factor (E_f_)

Loska et al. 2004 developed a modified technique for calculating EF based on the equation introduced^58^. Reference elements most frequently used are Sc, Mn, Ti, Al, Ca, Ni and Fe forms the basis of this technique^59–61^. The reference element used in this study was Ni. Nickel was chosen as the standard element because there is a lot of it in the Earth's crust, but the amount of it in soils varies depending on their matrices. As a reference environment that was comparable to I_geo_, the average elemental concentration in the Earth's crust was used.

2 $${\text{E}}_{{\text{f}}} = \, \left\{ {{\text{C}}_{{\text{n}}} \left( {{\text{sample}}} \right)/{\text{ C}}_{{{\text{ref}}}} \left( {{\text{sample}}} \right)} \right\} \, /\left\{ {{\text{B}}_{{\text{n}}} \left( {{\text{background}}} \right)/{\text{ B}}_{{{\text{ref}}}} \left( {{\text{background}}} \right)} \right\}$$

where B_n_ is the explored component, C_n_ is the analyzed element, C_ref_ is the standard component in the examined environment, and B_ref_ is the comparative baseline in the reference environment.

#### Contamination factor (C_f_)

The modified method for calculating the contamination factor was used in this study^62^. The contamination factor is calculated using the formula below.

3 $${\text{C}}_{{{\text{f}} }} = {\text{C}}_{{\text{o}}} /{\text{ C}}_{{\text{n}}}$$

Co represents the average metal content of at least five sample locations, and Cn represents the elemental concentration in the earth's crust. Hakanson classified contamination factor into four groups.

#### Potential ecological hazard index

Ecological Risk Assessment (ERA) methodology to evaluate the potential ecological risks posed by metals in soil and sediment^62^. In order to assess index-level risk, an ecological risk assessment investigates the toxicological, ecological, and environmental impacts of soil pollution and metals^63–65^. The ecological risk is calculated by using Eq. (4)

4 $${\text{E}}^{{\text{i}}}_{{\text{r}}} = {\text{ T}}^{{\text{i}}}_{{\text{r}}} \times \, \left( {{\text{C}}_{{\text{i}}} /{\text{C}}_{0} } \right)$$

where C_i_ is the sample metal concentration and C_0_ is the background metal concentration^56^. T^i^_r_ represents the toxic response factor of a specific metal. The toxic response factors (TRFs) for Cu (5), Cr (2), Pb (5), Zn (1), Ni (5), and As (10) were derived from the literature^62,66,67^. The risk (RI) values can be calculated by Eq. (5)

5 $${\text{RI}} = \mathop \sum \limits_{i = 0}^{n} E_{r}^{i}$$

On the basis of assessment, the Er values < 40, RI < 150 shows low ecological risk, 40 ≤ Er < 80, 150 ≤ RI < 300 shows moderate ecological risk, 80 ≤ Er < 160; 300 ≤ RI < 600 indicates considerable ecological risk, 160 ≤ Er < 320; RI ≥ 600 shows high ecological risk and Er ≥ 320 considered very high ecological risk.

### Human health risk assessment.

#### Exposure assessment

The health risk assessment is a reliable technique for estimating the harm presented to humans by contamination. Humans are often exposed to heavy metals in soils via three paths: (a) direct oral ingestion of sand grains; (b) dermal absorption of heavy metals in soil particles attached to exposed skin; and (c) mouth and nose inhaling of soil particles^68–80^. Calculations are performed using the following equations to determine the chronic daily dose (CDD: mg/kg/day) of potentially hazardous heavy metals absorbed via the three various exposure to non-carcinogenic heavy metals.

6 $$CDD\; Ingestion = \frac{C \times IRing \times ED \times EF}{{BW \times AT}} \times CF$$

7 $$CDD \;Inhalation = \frac{C \times IRing \times ED \times EF}{{BW \times AT \times PEF}}$$

8 $$CDD\; dermal = \frac{C \times SA \times SAF \times ED \times EF}{{BW \times AT}} \times CF$$

Soil ingestion was selected as the most preferred approach for humans exposed to environmentally hazardous components. Calculations and estimates of the chronic risk to the population of both adults and children (male and female) from soil intake were performed. In general, children have more direct contact with soil than adults, ingest more soil unintentionally, and are exposed to soil more often. In addition, it is believed that contaminants have a stronger influence on underweight children. Table 3 shows all of the exposure variables and values used to determine intake values and risk.

**Table 3:** Reference values of health risk parameters due to heavy metals in urban soils.

Heavy metals (mg/kg)	Reference doses (RfD)			Slope factors (SF)		
	Ingestion	Inhalation	Dermal	Ingestion	Inhalation	Dermal
As	3.00E − 04	1.23E − 04	1.23E − 04	1.50E + 00	4.30E − 03	3.66E + 00
Cr	3.00E − 04	2.86E − 05	3.00E − 03	5.01E − 01	4.20E + 01	2.00E + 01
Cu	4.00E − 02	4.00E − 02	1.20E − 02	–	–	–
Ni	2.00E − 02	2.06E − 02	5.40E − 03	1.70E + 00	–	4.25E + 01
Pb	1.40E − 03	3.52E − 03	5.24E − 04	8.50E − 03	–	–
Zn	3.00E − 01	0.30	6.00E − 02	–	–	–

#### Non-carcinogenic risk assessment

The hazard quotient (HQ) is a commonly used to measure for assessing non-carcinogenic risk. It is determined by dividing the chronic daily dose (CDD) by the reference dose (RfD) for a certain chemical^38,45^. The hazard index (HI) is the total of the HQ and means of the overall risk of non-carcinogenic components for a particular element based on three categories of risks. Following formulae determine HQ and HI.

9 $$HQ = \frac{CDD}{{RfD}}$$

10 $$HI = \sum HQi = \sum \frac{CDDi}{{RfDi}}$$

As per USEPA (1989), RfD is the guideline dose for each heavy metal as shown in Table 4. If the HI number is less than one, there is no threat of non-carcinogenic consequences occurring; if the HI value is higher than one, there is a possibility of potential non-carcinogenic impacts on health.

**Table 4:** Reference doses (RfD) for non-carcinogenic heavy metals and slope factors (SF) for carcinogenic heavy metals.

Acronym^ref^	Risk parameter	Unit	Adults		Children	
			Male	Female	Male	Female
IR_ing_^73^	Ingestion rate of soil	mg/day	50	50	100	100
ED^76^	Exposure duration	Years	30	30	6	6
EF^77^	Exposure frequency	days/year	350	350	350	350
CF^73^	Conversion factor	kg/mg	1 × 10^–6^	1 × 10^–6^	1 × 10^–6^	1 × 10^–6^
BW^69, 70^	Body weight of the exposed individual	Kg	62.8	54.7	17.2	16.5
AT^73^	Average time	Years	10,500	10,500	2100	2100
SA^71^	Exposed skin surface area	Cm^2^	2120	1910	1090	1060
SAF^77^	Skin adherence factor	mg/cm^2^	0.07	0.07	0.2	0.2
DAF^78^	Dermal absorption factor	–	0.13	0.13	0.13	0.13
IR_inh_^72^	Inhalation rate of soil	m^3^/day	13.1	11.3	7.6	7.4
PEF^78^	Particle emission factor	m^3^/kg	1.4 × 10^9^	1.4 × 10^9^	1.4 × 10^9^	1.4 × 10^9^

#### Carcinogenic risk assessment

Carcinogenic risk is the likelihood that a human may acquire any sort of cancer over the course of their life owing to exposure to carcinogenic risks^38,74,75,81^. The following formula is used to evaluate the lifetime carcinogenic health hazards associated with a particular heavy metal^45,76^.

11 $${\text{CR }} = {\text{ CDD }} \times {\text{ SF}}$$

12 $${\text{TCR }} = \, \sum {\text{ CR}}$$

where CR denotes the carcinogenic risk, TCR represents the total carcinogenic risk, and SF indicates the slope factor (mg/kg/day) as shown in Table 4. The US Environmental Protection Agency (1989) suggests that a CR and TCR value of less than 1 × 10^−6^ should be considered inconsequential, whereas a CR and TCR value that exceeds 1 × 10^−6^is considered to be hazardous to human health.

## Results and discussion

### Descriptive statistical summary of heavy metals in soils

The obtained elemental concentrations with descriptive statistical variables for the six heavy metals (As, Cr, Cu, Ni, Pb, and Zn) employed in this study together with background values^57^ and Canadian soil quality guidelines values^82^ are shown in Table 5. In the soil concentrations for individual heavy metals, ranges showed 0.10 to 15.9 mg/kg for As, 2.94 to 707.1 mg/kg for Cr, 6.86 to 2324 mg/kg for Cu, 14.27 to 234.0 mg/kg for Ni, 9.03 to 1664.5 mg/kg for Pb, and 60.7 to 961.8 mg/kg for Zn, with average concentrations of 3.2, 203.3, 253.3, 73.1, 68.6, and 229.6 (mg/kg) respectively. For evaluating contamination in the study area, background soil concentrations of the examined components are essential. The mean concentrations of As, Cr, Cu, Ni, Pb, and Zn largely exceeded Taylor and McLennan 1995, but when compared to Canadian soil quality guidelines Cr, Cu, and Ni metals exceeded guideline values, and the remaining metals were within the limits, indicating that surface soils are greatly polluted by these heavy metals. Produced water is usually formation water from oil and gas extraction. It may also contain dissolved inorganic salts, hydrocarbons, minerals, trace metals, naturally occurring radioactive substances, production chemicals, and dissolved gases^83–86^. This phenomenon is attributable to the extensive use of chemical substances containing high concentrations of heavy metals discharged into the environment due to petroleum drilling and extraction. The effects of petroleum exploration and production operations on the heavy metal contents of soil and groundwater in the Niger Delta^87^ and also, one of the investigations revealed that the soil in close proximity to sites where oil and gas drilling waste is discharged may be influenced by elevated levels of certain heavy metals^88^. The present work was compared with similar studies carried out in other countries like Pakistan^88^, Iran^89,90^ and India^91^ (Table 6).

**Table 5:** Descriptive statistical summary of heavy metal concentrations in soils and Reference values.

Parameters	As (mg/kg)	Cr (mg/kg)	Cu (mg/kg)	Ni (mg/kg)	Pb (mg/kg)	Zn (mg/kg)
Mean	3.2	203.3	253.3	73.1	68.6	229.6
Minimum	0.10	2.9	6.8	14.2	9.0	60.7
Maximum	15.9	707.1	2324.1	234.0	1664.5	961.8
Std. Deviation	4.0	108.1	347.4	22.8	153.0	137.6
Skewness	1.8	2.3	3.4	2.5	8.6	2.5
Kurtosis	1.8	7.0	13.4	20.2	87.3	9.9
Reference value^57^	1.5	35.0	25.0	20.0	20.0	71.0
Reference value^82^	12	64	63	45	70	250

**Table 6:** Heavy metal s mean concentrations (mg/kg) in soils comparison of similar studies with the present work.

Study area	As	Cr	Cu	Ni	Pb	Zn	Cited ^Reference No^
Iran	NA	0.028	1.146	1.150	1.458	1.679	^89^
Pakistan	NA	163.7	NA	11.603	1015.7	402.4	^88^
India	NA	8.29	13.52	18.78	12.52	NA	^91^
Iran	6.2	60	97	84	43	165	^90^
India	3.2	203.3	253.3	73.1	68.6	229.6	Present study area

### Spatial distribution maps

**Figure 3:**
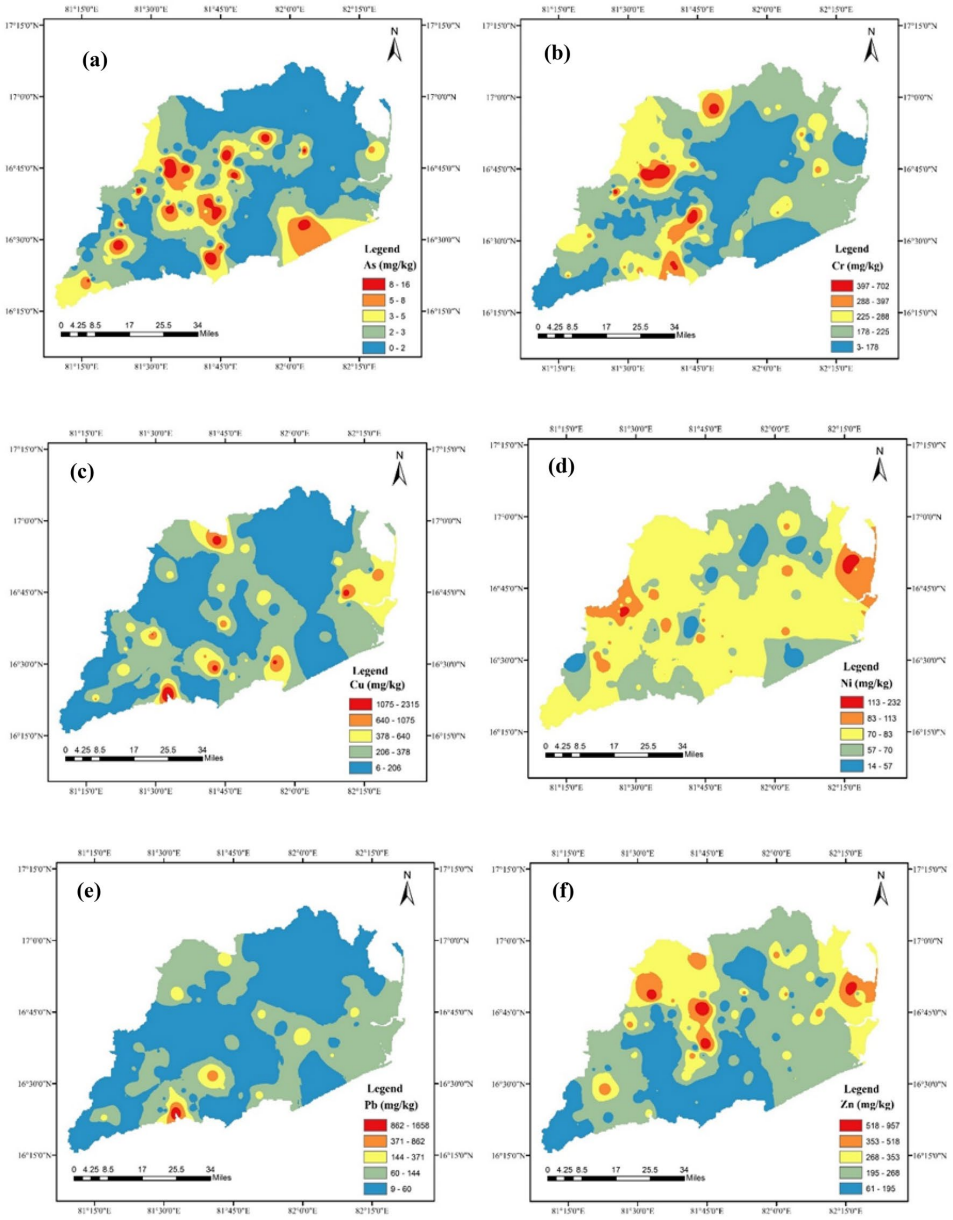
Spatial distribution of heavy metals (**a**) As;(**b**) Cr; (**c**) Cu; (**d**) Ni; (**e**) Pb; (**f**) Zn in soils. (maps were generated with software ArcGIS 10.7 http://www.esri.com).

Heavy metal concentration maps are an extremely useful tool for delineating zones with significant metal contents in the study area. The spatial distribution patterns of As, Cr, Cu, Ni, Pb, and Zn (Fig. 3) in the study area's top soils were prepared. Samples taken near drilling sites revealed the highest amounts of all harmful elements. Diverse concentrations of the six constituent elements were observed across different drilling regions, suggesting a degree of dispersion of hazardous elements due to various extraction processes and soil degradation mechanisms. The distribution of As towards west side of the study area indicates the probable source would be from agriculture activity due to usage of pesticides. Cu, Pb, Ni and Cr are observed to be exceeding the reference values given by Canadian guideline and Taylor McLennan values. This is mainly observed nearby drilling sites, with major source from back water containing various inorganic compounds and thereby getting deposited on to the surface. Overall the distribution of heavy metals is concentrated towards south-west of the study area due to major drilling activity, when compared to north-east where the drilling activity is minimal.

### Index of geo-accumulation (I_geo_)

**Figure 4:**
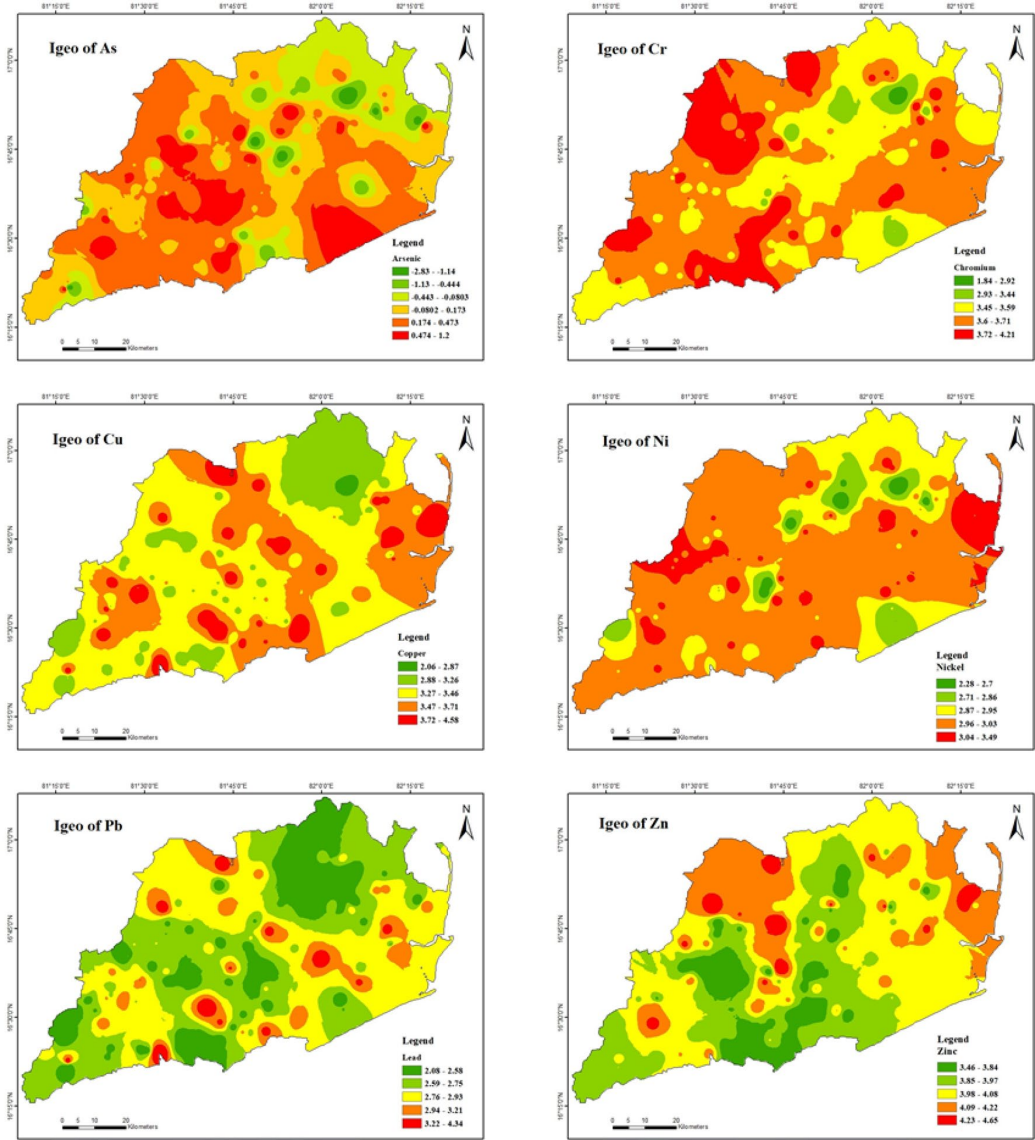
Spatial distribution of geo accumulation index of heavy metals in soils (maps were generated with software ArcGIS 10.7 http://www.esri.com).

The concentrations of the six heavy metals in the soils were calculated (Table 7) using the Igeo index (Fig. 4) The study area exhibited a wide range of contamination levels, ranging from pristine to highly contaminated. The I_geo_ value for As ranged from − 2.83 to 1.2 and was greater than 0 for 82% of the soil samples, classifying them as slightly to moderately polluted. The Igeo values of Cr, Cu, Ni, Pb, and Zn range from 1.83 to 4.21, 2.05 to 4.58, 2.27 to 4.34, 2.08 to 4.34, and 3.45 to 4.65, with 100% of soil samples having an Igeo grade greater than 2, respectively, indicating these heavy metals are moderately to heavily contaminated. Reports indicate the presence of arsenic contamination in groundwater, soil, and food in different areas of India, such as Uttar Pradesh, Jharkhand, Bihar, Madhya Pradesh, Chhattisgarh, Assam, Manipur, Tripura, Arunachal Pradesh, Punjab, and Andhra Pradesh^1,92–98^.

**Table 7:** Pollution indices (Igeo, EF & CF) for heavy metals in soils.

Pollution indices	Elements	Mean	Maximum	Minimum	% of samples exceeding limits						
					< 0	0 < 1	1 < 2	2 < 3	3 < 4	4 < 5	> 5
I_geo_	As	0.1	1.2	− 2.8	18	69	13	0	0	0	0
	Cr	3.6	4.2	1.8	0	0	0.7	0	93.6	5.7	0
	Cu	3.4	4.5	2.0	0	0	0	2	88	10	0
	Ni	2.9	3.4	2.2	0	0	0	60.5	39.5	0	0
	Pb	2.7	4.3	2.0	0	0	0	80.5	18.8	0.7	0
	Zn	3.9	4.6	3.4	0	0	0	0	53.3	46.7	0

	Elements	Minimum	Maximum	Mean	% of samples exceeding limits						
					< 2	2–5	5–20	20–40	> 40		
EF	As	0.0	8.1	0.7	89.9	7.1	3	0	0		
	Cr	0.1	4.9	1.6	74.8	25.2	0	0	0		
	Cu	0.3	28.7	2.9	69	16.5	13	1.5	0		
	Ni	1	1	1	100	0	0	0	0		
	Pb	0.0	25.7	1.0	89	9.6	0.7	0.7	0		
	Zn	0.2	4.9	0.9	95	5	0	0	0		

	Elements	Minimum	Maximum	Mean	% of samples exceeding limits						
					< 1	1 < 3	3 < 6	> 6			
CF	As	0.0	10.6	2.1	39.5	41.7	3.5	15.1			
	Cr	0.0	20.2	5.8	0.7	5.7	61.8	31.6			
	Cu	0.0	9.1	0.9	74.8	17.9	5.7	1.4			
	Ni	0.7	11.7	3.6	1.4	10	87	1.4			
	Pb	0.4	83.2	3.4	13.6	64	9.3	12.9			
	Zn	0.8	13.5	3.2	2.1	51.7	41.7	4.3

### Enrichment factor (EF)

**Figure 5:**
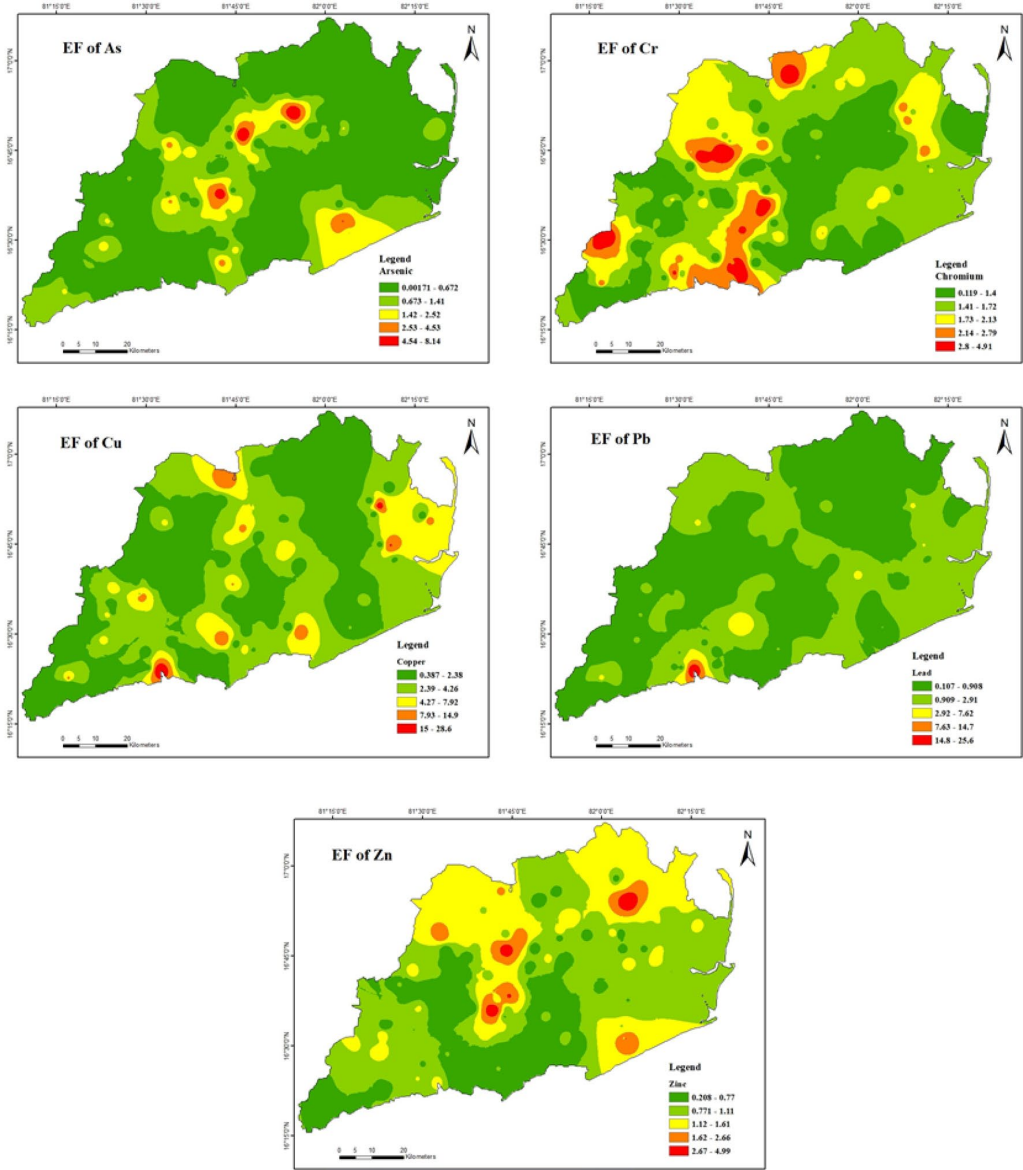
Spatial distribution of enrichment factor of heavy metals in soils (maps were generated with software ArcGIS 10.7 http://www.esri.com).

The enrichment factor was used to determine soil heavy metal concentrations (Fig. 5). Table 7 shows that the EF values for Cr, and Zn in soils varied from 0.1 to 4.9, and 0.2 to 4.9, with 25.2%, and 5% respectively, of soil samples classified as having indicating deficiency to minor enrichment. However, the EF of As, Cu and Pb ranged from 0.0 to 8.1, 0.3 to 28.7 and 0.0 to 25.7 with 10.1%, 31% and 11% respectively, of soil samples falling into moderate to significant enrichment. The maximum levels documented for arsenic, copper, and lead were obtained from a solitary low-capacity produced water source from a gas and condensate field. This study examines the environmental effects of the Norwegian offshore petroleum industry's discharge of produced water and drilling waste^86^.

### Contamination factor (CF)

**Figure 6:**
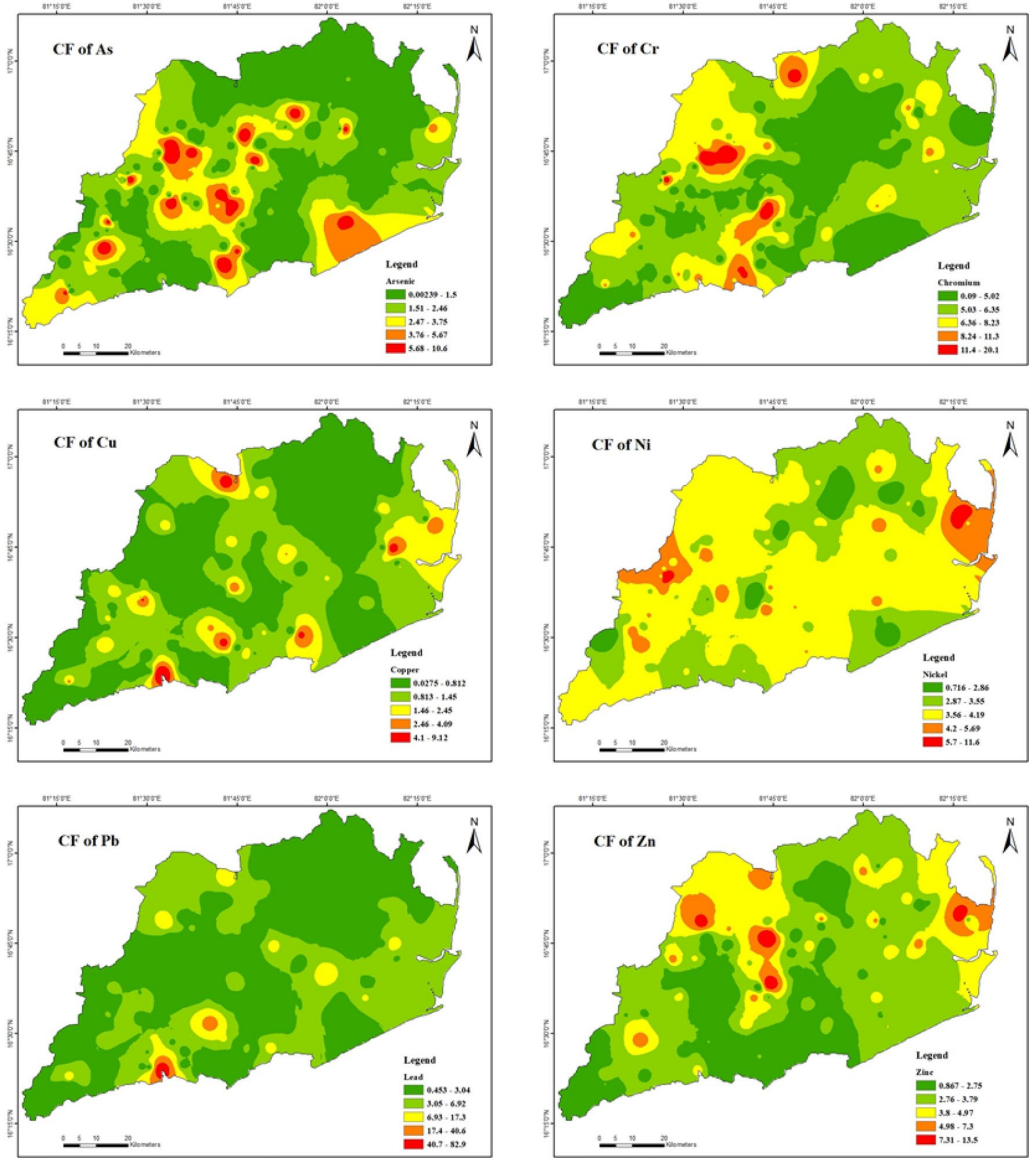
Spatial distribution of contamination factor of heavy metals in soils (maps were generated with software ArcGIS 10.7 http://www.esri.com).

Using the contamination factor (Fig. 6), the levels of the six different heavy metals calculated in the soil were determined. Based on calculations in Table 7, the mean CF values observed for As and Cu in soils were 2.1 and 0.9, respectively. The coefficient of variation (CF) values for the elements above exhibited a range of 0.0 to 10.6 and 0.0 to 9.1, respectively, with 60.3% and 25% of soils suggesting minimal to high contamination. The contamination factor (CF) of Cr, Ni, Pb, and Zn ranged from 0.0 to 20.2, 0.7 to 11.7, 0.4 to 83.2, and 0.8 to 13.5, respectively, with 97.3%, 98.6%, 86.4%, and 97.9% of soil CF grade values indicating extremely contaminated soil. The elevated concentrations of Cu, Pb, and Zn observed in the study area are primarily attributed to human activities, particularly emissions via sediment waste deposition through back water effluents^99–102^.

### Principle component analysis

Principal Component Analysis (PCA) was performed to study the complex linear correlation between metal concentrations in soils, which enables the interpretation of correlation of elements in the study area. Elements belonging to a given factor were defined by factor matrix after varimax rotation, with those having strong correlations grouped into factors. The aforementioned multielement factors were split into two categories based on the influence they had on the surface soils by determining the distribution of elements in the study area: (1) factors with significant anthropogenic influence, (2) factors that are primarily the result of anthropogenic effects or other natural processes. However, factors are identified based on dominant influence. The distribution of individual association of elements in soil was determined by principal component method (as shown in Table 8). Based on the eigen values and varimax rotation, As, Cr, Cu, Ni, Pb and Zn concentrations could be grouped into a three component model, which accounted for 73.20% of all the data variation. The first component matrix (PC1) displayed high values for Cu and Pb indicating their association, second component (PC2) showing Cr and Ni, whereas third component (PC3) displayed high Zn concentrations. Factor PC1 explains 73.2% of total variance with positive loading on Cu and Pb (r > 0.90). This factor can be attributed to anthropogenic influence of these heavy metals, probably as a result of back water or produced water from the drilling rigs, which contain various inorganic chemicals with these metals as by-products. The high values of Cu and Pb are observed in the old and active drilling sites at Mandapeta and Bantumilli of the study area. Further, the Pb being one of the major chemicals among others like water, sand, salt, citric acid this component can be attributed to anthropogenic. The chemicals that gas companies use include water. Factor PC2 showed Cr and Ni as significant loadings which explained 52.85% of the overall variation. The positive loadings of Cr (r = 0.779) and Ni (r = 0.820) indicate the source to be attributed as anthropogenic/geogenic. This is observed in Nagendrapuram and Arjunudupalem drilling sites of the study area. Further, the source of Cr could be a contaminant in the chemical stew used in fracking fluids, but it also occurs naturally in the ground, so the possibility exists that it might picked up there by fracking fluids pumped into deep gas wells under enormous pressure and then discarded after the drilling is complete. Whereas, the source of Ni would be from the chemicals used in fracking fluids where Nickle sulphate being one of the inorganic compound. PC3 represented 28.68% of the total variance and loadings on Zn with positive loading (r = 0.739), observed in Prathallamaraka drilling site.

**Table 8:** Total variance explained and component matrices for the heavy metals.

Component loadings	Initial eigenvalues			Extraction sums of squared loadings			Rotation sums of squared loadings		
	Total	% of Variance	Cumulative %	Total	% of variance	Cumulative %	Total	% of variance	Cumulative %
1	1.816	30.269	30.269	1.816	30.269	30.269	1.721	28.683	28.683
2	1.444	24.065	54.334	1.444	24.065	54.334	1.450	24.166	52.850
3	1.133	18.875	73.209	1.133	18.875	73.209	1.222	20.359	73.209
4	0.761	12.675	85.884						
5	0.573	9.548	95.433						
6	0.274	4.567	100.000						

Elements	Rotated component matrix								
	PC1	PC2	PC3						
As	− 0.022	0.288	− 0.73						
Cr	0.092	**0.779**	− 0.265						
Cu	**0.910**	− 0.015	0.119						
Ni	− 0.067	**0.820**	0.229						
Pb	**0.926**	0.039	0.031						
Zn	0.146	0.291	**0.739**						

#### Assessment of potential ecological risk

**Figure 7:**
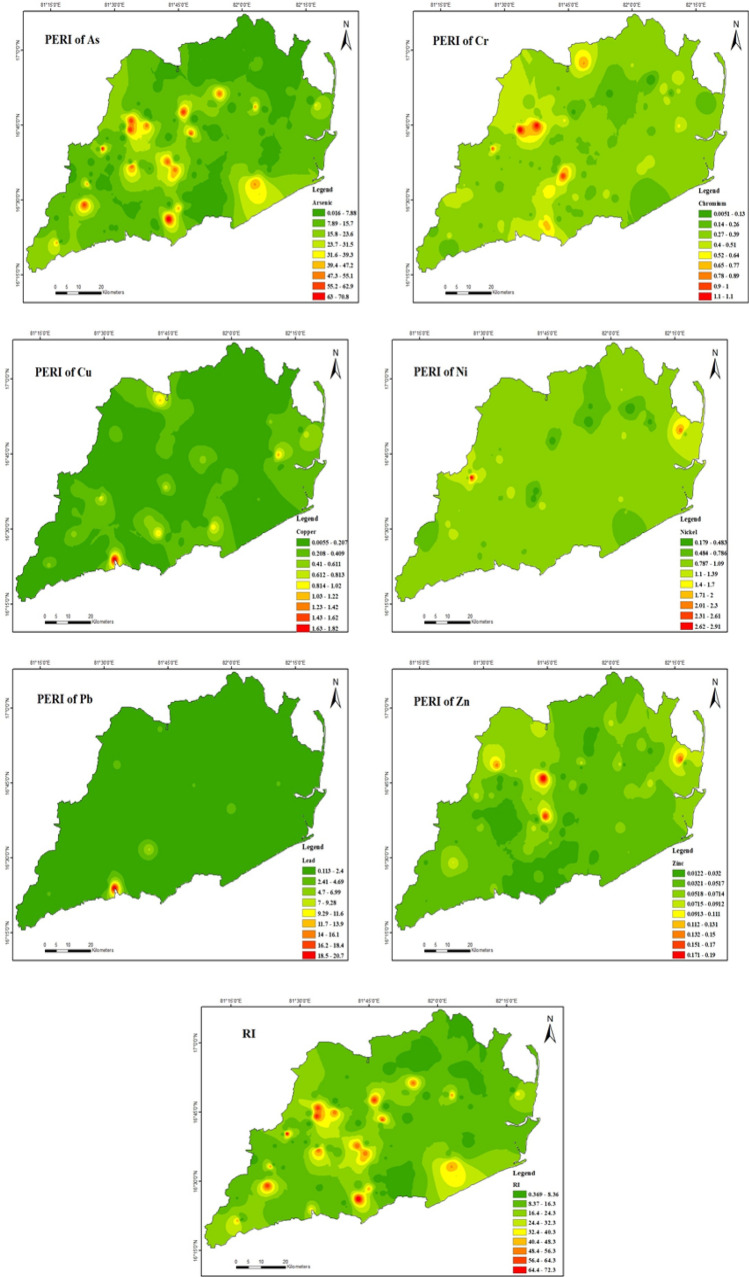
Spatial distribution of Potential Ecological Risk Index (PERI) & Risk Index (RI) (maps were generated with software ArcGIS 10.7 http://www.esri.com).

The potential ecological risk index (PERI) values of six different heavy metals discovered (Table 9) in the area of the study's topsoil were calculated. All of the elements As, Cr, Cu, Ni, Pb, and Zn (Fig. 7) exhibited mean values of Efi that were lower than 40, with mean values in the order As (14.2) > Ni (0.9) > Pb (0.8) > Cr (0.3) > Cu (0.1) > Zn (0.04) representing that these metals posed a low potential ecological risk. It is important to note, however, that several of the sampling sites revealed a potential ecological risk that ranged from moderate especially for arsenic, the maximum value was 0.0 to 70.9. In contrast As is much complex than those of the other heavy metals, 15% of all samples for arsenic demonstrated a moderate potential ecological risk level. Similar to this, many authors in their studies proposed that using arsenic-enriched fertilizers and pesticides or irrigating crops with arsenic-contaminated water could lead to higher levels of arsenic in soil^49,103^. Few mining areas in China observed in PERI results that As metal showed moderate potential ecological risk and suggested that As is likely derived from other sources such as mining and related industrial activities^67^. The results of RI ranged in between 0.3 and 72.3, with a mean of 16.6, indicating that six heavy metals of soils of the study area were in low risk category (Table 9).

**Table 9:** Potential ecological risk index (PERI and RI) values of heavy metals.

Elements	Minimum	Maximum	Mean
As	0.0065	70.9198	14.2749
Cr	0.0048	1.1545	0.3321
Cu	0.0054	1.8301	0.1995
Ni	0.1783	2.9259	0.9142
Pb	0.1128	20.8070	0.8581
Zn	0.0121	0.1908	0.04456
RI	0.357979	72.37594	16.62427

#### Non-carcinogenic health risks

Non-carcinogenic health risk assessments in surface soils were performed on As, Cr, Cu, Ni, Pb, and Zn using primary exposures: ingestion (CDD ingestion), dermal (CDD dermal), and inhalation (CDD inhalation) for both adults and children (male and female). The HQ of adults and children due to three exposure routes decreased as follows: Ingestion > Dermal > Inhalation.

**Figure 8:**
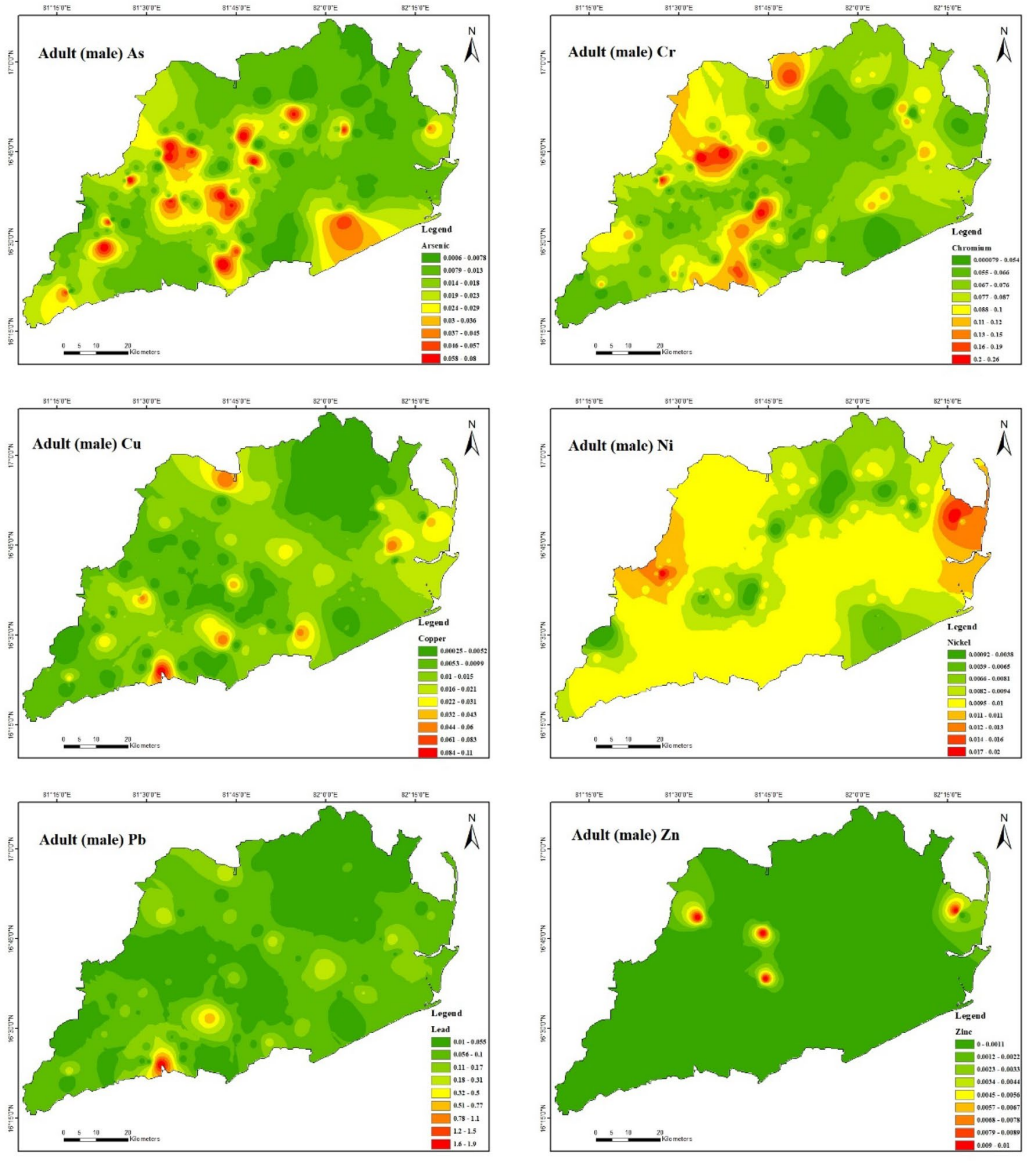
Spatial distribution of non-carcinogenic health risk due to heavy metals in Adult (male) (maps were generated with software ArcGIS 10.7 http://www.esri.com).

**Figure 9:**
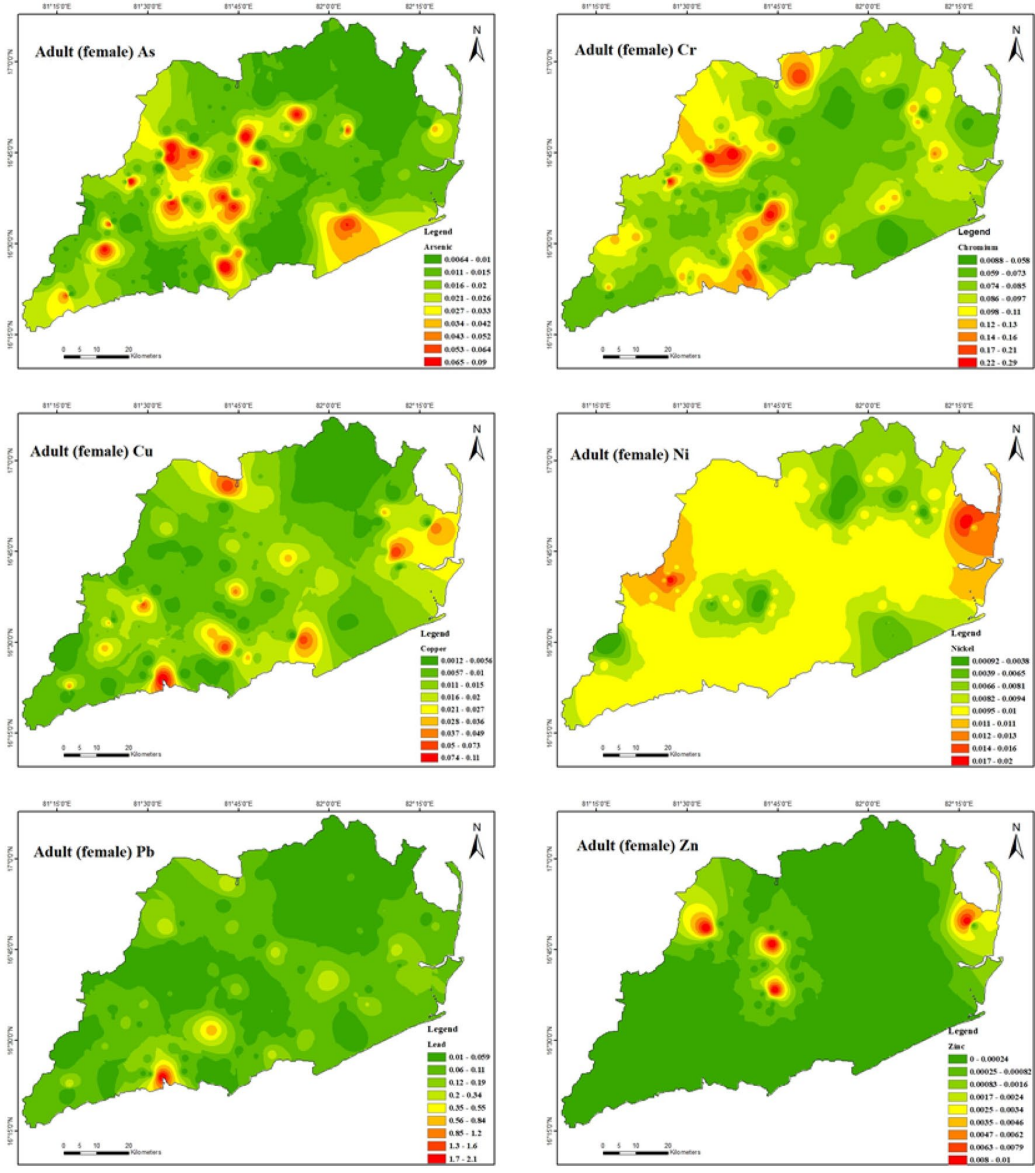
Spatial distribution of non-carcinogenic health risk due to heavy metals in Adult (female) (maps were generated with software ArcGIS 10.7 http://www.esri.com).

Furthermore, demonstrates that the non-carcinogenic health consequences of exposure to heavy metals for male adults (Fig. 8) via ingestion are 3.9E − 06 to 4.2E − 02 for As, 7.8E − 04 to 1.9E − 01 for Cr, 1.4E − 04 to 4.6E − 02 for Cu, 5.7E − 04 to 9.3E − 03 for Ni, 5.1E − 03 to 9.5E − 01 for Pb and 1.6E − 04 to 2.6E − 03 for Zn. In this instance, the hazard quotient (HQ) of Pb was extensively greater compared to other heavy metals due to its higher content in the soil in the study area. However, the estimated hazard index (HI) values for Pb ranged from 1.0E − 02 to 1.9E + 00 for male adults, indicating that Pb had some negative impacts on male Adults in the study region. Table 10 presents the non-carcinogenic health effects of heavy metal exposure through ingestion for adult females (Fig. 9). The results indicate that the range of health consequences for As is 4.5E − 06 to 4.9E − 02, for Cr is 9.0E − 04 to 2.2E − 01, for Cu is 1.6E − 04 to 5.3E − 02, for Ni is 6.5E − 04 to 1.1E − 02, for Pb is 5.9E − 03 to 1.1E + 00, and for Zn is 1.9E − 04 to 2.9E − 03. The hazard quotient (HQ) of chromium (Cr) and lead (Pb) was found to be significantly higher than that of other heavy metals in the study area due to their elevated concentrations in the soil. The study findings suggest the hazard index (HI) values for Cr and Pb were estimated to range from 1.2E − 03 to 2.9E − 01 and 1.1E − 02 to 2.1E + 00, respectively, for female adults. These results indicate that Cr and Pb in the study region may adversely affect female adults. For both male and female adults in the study region, the HI was found to be in the following order: Pb > Cr > As > Cu > Ni > Zn. It appears there is no considerable danger to male adult health from the non-carcinogenic effects of As, Cr, Cu, Ni, and Zn and to female adult health from the non-carcinogenic effects of As, Cu, Ni, and Zn in the study area.

**Figure 10:**
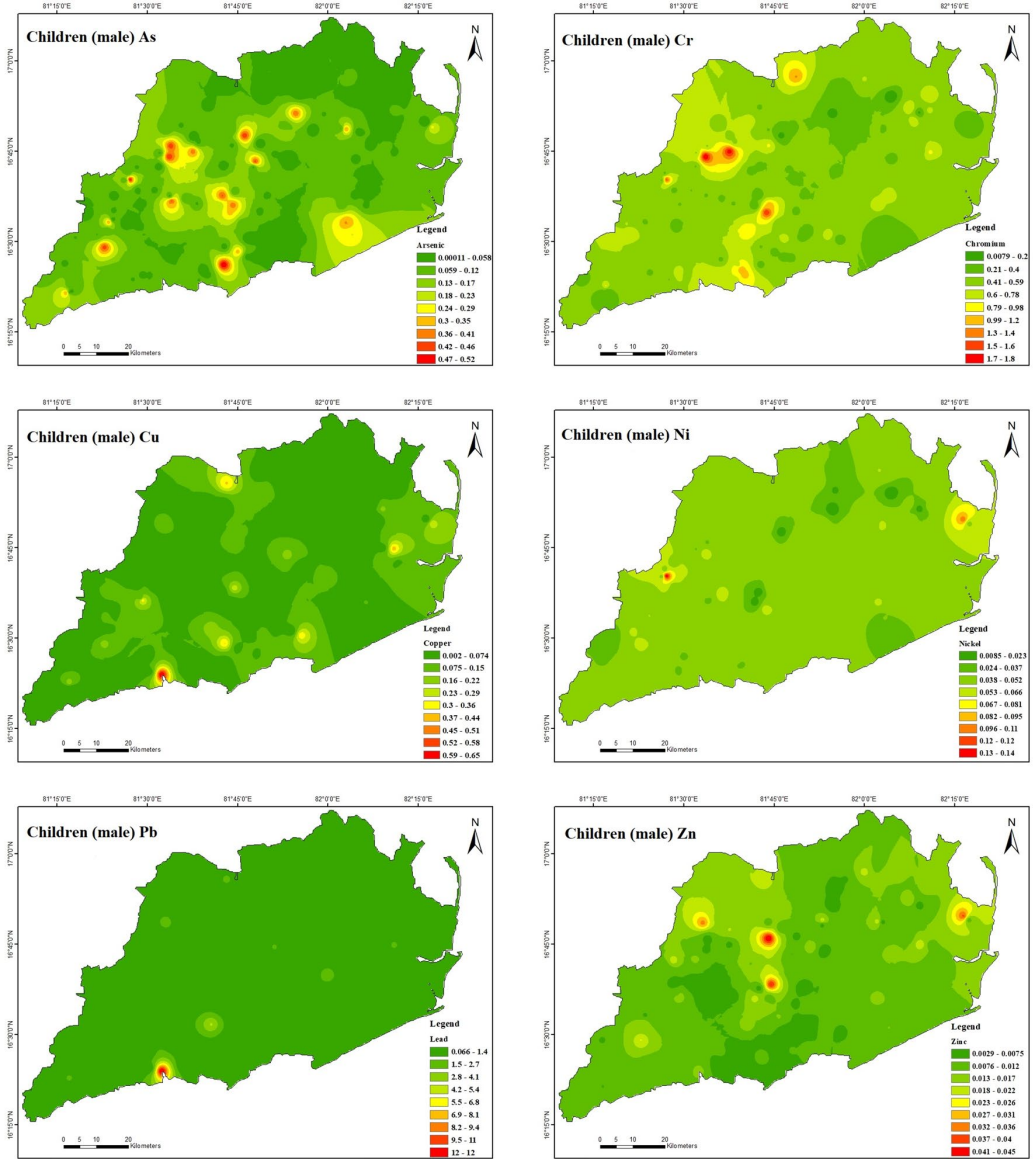
Spatial distribution of non-carcinogenic health risk due to heavy metals in. Children (male) (maps were generated with software ArcGIS 10.7 http://www.esri.com).

**Figure 11:**
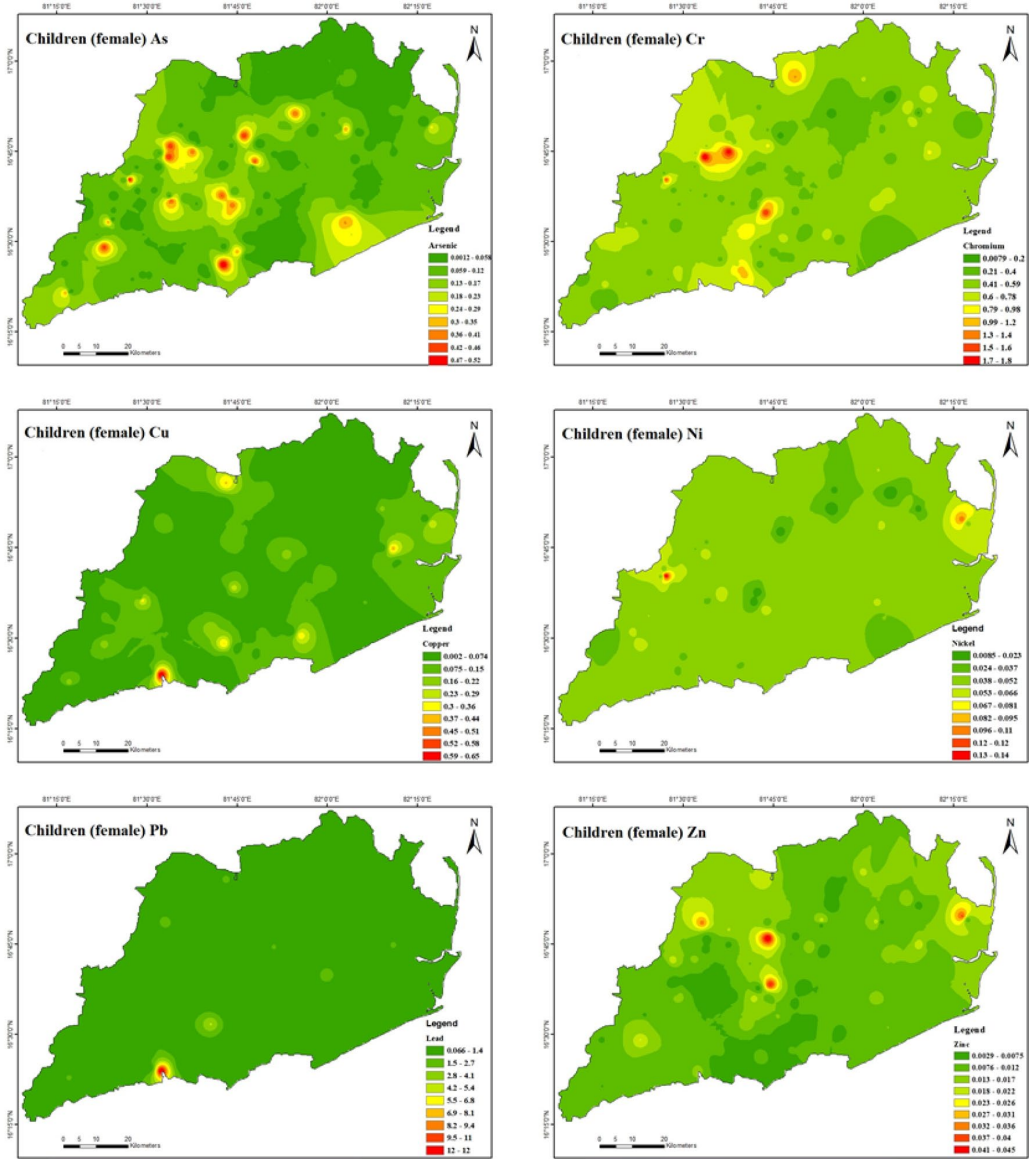
Spatial distribution of non-carcinogenic health risk due to heavy metals in Children (female) (maps were generated with software ArcGIS 10.7 http://www.esri.com).

Table 10 further indicates that the non-carcinogenic health effects of heavy metal exposure for male children (Fig. 10) through ingestion are 2.8E − 05 to 3.1E − 01 for As, 5.7E − 03 to 1.4E + 00 for Cr, 1.0E − 03 to 3.4E − 01 for Cu, 4.1E − 03 to 6.8E − 02 for Ni, 3.7E − 02 to 6.9E + 00 for Pb, and 1.2E − 03 to 1.9E − 02 for Zn. The present study reveals that the hazard quotient (HQ) of chromium (Cr) and lead (Pb) was significantly higher in comparison to other heavy metals because of their significantly raised levels in the soil of the investigated region. The study observations reveal that the estimated hazard index (HI) values for Cr and Pb ranged from 7.4E − 03 to 1.8E + 00 and 6.6E − 02 to 1.2E + 01, respectively, for male children. These results suggest that Cr and Pb may adversely affect male adults in the study area. Also, it has been demonstrated that ingesting heavy metals can result in non-carcinogenic health effects for female children (Fig. 11). Specifically, the estimated health consequences for exposure to heavy metals via ingestion are 3.0E − 05 to 3.2E − 01 for As, 5.9E − 03 to 1.4E + 00 for Cr, 1.0E − 03 to 3.5E − 01 for Cu, 4.3E − 03 to 7.1E − 02 for Ni, 3.9E − 02 to 7.2E + 00 for Pb, and 1.2E − 03 to 1.9E − 02 for Zn. The increased Cr and Pb levels in the soil in the study area resulted in a significantly higher hazard quotient (HQ) than other heavy metals. For female children, the estimated hazard index (HI) values for Cr varied from 7.6E − 03 to 1.8E + 00 and Pb from 6.7E − 02 to 1.2E + 01, demonstrating that Cr and Pb had some adverse effects in the research area. For both male and female children in the study region, the HI was found to be in the following order: Cr > Pb > As > Cu > Ni > Zn. It appears there is no considerable risk to male and female children health from the non-carcinogenic effects of As, Cu, Ni, and Zn in the study area because the HI values are all lower than the safe limit (HI =  < 1) for all sampling points. The study found that the amounts of HQ exposed to adults and children via skin contact and inhalation were substantially lower than those exposed to HQ through ingestion, indicating that these pathways pose no health concern. This indicates that the greater concentration of soils in the study area indicates that the hazard quotient (HQ) of Cr and Pb is higher than for other heavy metals. The non-carcinogenic risk level distributions of the study area were mapped, and which Cr and Pb exhibited the most severe scenarios were observed. The non-carcinogenic risk values of both adults and children were used in the present study. It indicates that the non-carcinogenic risk values in provinces surrounded by drilling rigs are comparatively higher than in various other areas.

**Table 10:** Results of non-carcinogenic risks through ingestion, inhalation, and dermal pathways for adults and children (male and female).

	Non-carcinogenic	Adult male				Adult female			
		HQ_ing_	HQ_inh_	HQ_der_	HI	HQ_ing_	HQ_inh_	HQ_der_	HI
As	Minimum	3.9E − 06	1.8E − 09	3.6E − 06	7.5E − 06	4.5E − 06	1.8E − 09	3.8E − 06	8.2E − 06
	Maximum	4.2E − 02	1.9E − 05	4.0E − 02	8.2E − 02	4.9E − 02	1.9E − 05	4.1E − 02	9.0E − 02
	Mean	8.5E − 03	3.9E − 06	8.0E − 03	1.7E − 02	9.8E − 03	3.9E − 06	8.3E − 03	1.8E − 02
Cr	Minimum	7.8E − 04	1.5E − 05	3.0E − 04	1.1E − 03	9.0E − 04	1.5E − 05	3.1E − 04	1.2E − 03
	Maximum	1.9E − 01	3.7E − 03	7.2E − 02	2.6E − 01	2.2E − 01	3.6E − 03	7.5E − 02	2.9E − 01
	Mean	5.4E − 02	1.1E − 03	2.1E − 02	7.6E − 02	6.2E − 02	1.0E − 03	2.2E − 02	8.5E − 02
Cu	Minimum	1.4E − 04	2.6E − 08	1.8E − 04	3.1E − 04	1.6E − 04	2.5E − 08	1.8E − 04	3.4E − 04
	Maximum	4.6E − 02	8.7E − 06	5.9E − 02	1.1E − 01	5.3E − 02	8.6E − 06	6.2E − 02	1.1E − 01
	Mean	5.0E − 03	9.4E − 07	6.5E − 03	1.2E − 02	5.8E − 03	9.3E − 07	6.7E − 03	1.2E − 02
Ni	Minimum	5.7E − 04	1.0E − 07	8.1E − 04	1.4E − 03	6.5E − 04	1.0E − 07	8.4E − 04	1.5E − 03
	Maximum	9.3E − 03	1.7E − 06	1.3E − 02	2.3E − 02	1.1E − 02	1.7E − 06	1.4E − 02	2.4E − 02
	Mean	2.9E − 03	5.3E − 07	4.2E − 03	7.1E − 03	3.3E − 03	5.2E − 07	4.3E − 03	7.6E − 03
Pb	Minimum	5.1E − 03	3.8E − 07	5.3E − 03	1.0E − 02	5.9E − 03	3.8E − 07	5.5E − 03	1.1E − 02
	Maximum	9.5E − 01	7.0E − 05	9.8E − 01	1.9E + 00	1.1E + 00	7.0E − 05	1.0E + 00	2.1E + 00
	Mean	3.9E − 02	2.9E − 06	4.0E − 02	7.9E − 02	4.5E − 02	2.9E − 06	4.2E − 02	8.6E − 02
Zn	Minimum	1.6E − 04	3.0E − 08	3.1E − 04	4.7E − 04	1.9E − 04	3.0E − 08	3.2E − 04	5.1E − 04
	Maximum	2.6E − 03	4.8E − 07	4.9E − 03	7.5E − 03	2.9E − 03	4.7E − 07	5.1E − 03	8.0E − 03
	Mean	6.1E − 04	1.1E − 07	1.2E − 03	1.8E − 03	7.0E − 04	1.1E − 07	1.2E − 03	1.9E − 03

	Non-carcinogenic	Children male				Children female			
		HQ_ing_	HQ_inh_	HQ_der_	HI	HQ_ing_	HQ_inh_	HQ_der_	HI
As	Minimum	2.8E − 05	3.7E − 09	2.0E − 05	4.8E − 05	3.0E − 05	3.8E − 09	1.9E − 05	4.9E − 05
	Maximum	3.1E − 01	4.1E − 05	2.1E − 01	5.2E − 01	3.2E − 01	4.2E − 05	2.1E − 01	5.3E − 01
	Mean	6.2E − 02	8.2E − 06	4.3E − 02	1.1E − 01	6.5E − 02	8.4E − 06	4.2E − 02	1.1E − 01
Cr	Minimum	5.7E − 03	3.2E − 05	1.6E − 03	7.4E − 03	5.9E − 03	3.3E − 05	1.6E − 03	7.6E − 03
	Maximum	1.4E + 00	7.8E − 03	3.9E − 01	1.8E + 00	1.4E + 00	7.9E − 03	3.8E − 01	1.8E + 00
	Mean	3.9E − 01	2.2E − 03	1.1E − 01	5.1E − 01	4.1E − 01	2.3E − 03	1.1E − 01	5.2E − 01
Cu	Minimum	1.0E − 03	5.4E − 08	9.4E − 04	1.9E − 03	1.0E − 03	5.5E − 08	9.2E − 04	2.0E − 03
	Maximum	3.4E − 01	1.8E − 05	3.2E − 01	6.6E − 01	3.5E − 01	1.9E − 05	3.1E − 01	6.6E − 01
	Mean	3.7E − 02	2.0E − 06	3.5E − 02	7.2E − 02	3.8E − 02	2.0E − 06	3.4E − 02	7.2E − 02
Ni	Minimum	4.1E − 03	2.2E − 07	4.4E − 03	8.5E − 03	4.3E − 03	2.2E − 07	4.2E − 03	8.6E − 03
	Maximum	6.8E − 02	3.6E − 06	7.1E − 02	1.4E − 01	7.1E − 02	3.6E − 06	6.9E − 02	1.4E − 01
	Mean	2.1E − 02	1.1E − 06	2.2E − 02	4.4E − 02	2.2E − 02	1.1E − 06	2.2E − 02	4.4E − 02
Pb	Minimum	3.7E − 02	8.1E − 07	2.8E − 02	6.6E − 02	3.9E − 02	8.2E − 07	2.8E − 02	6.7E − 02
	Maximum	6.9E + 00	1.5E − 04	5.2E + 00	1.2E + 01	7.2E + 00	1.5E − 04	5.1E + 00	1.2E + 01
	Mean	2.9E − 01	6.2E − 06	2.2E − 01	5.0E − 01	3.0E − 01	6.2E− 06	2.1E − 01	5.1E − 01
Zn	Minimum	1.2E − 03	6.4E − 08	1.7E − 03	2.8E − 03	1.2E − 03	6.5E − 08	1.6E − 03	2.9E − 03
	Maximum	1.9E − 02	1.0E − 06	2.6E − 02	4.5E − 02	1.9E − 02	1.0E − 06	2.6E − 02	4.5E − 02
	Mean	4.5E − 03	2.4E − 07	6.3E − 03	1.1E − 02	4.6E − 03	2.5E − 07	6.1E − 03	1.1E − 02

#### Carcinogenic health risks

**Figure 12:**
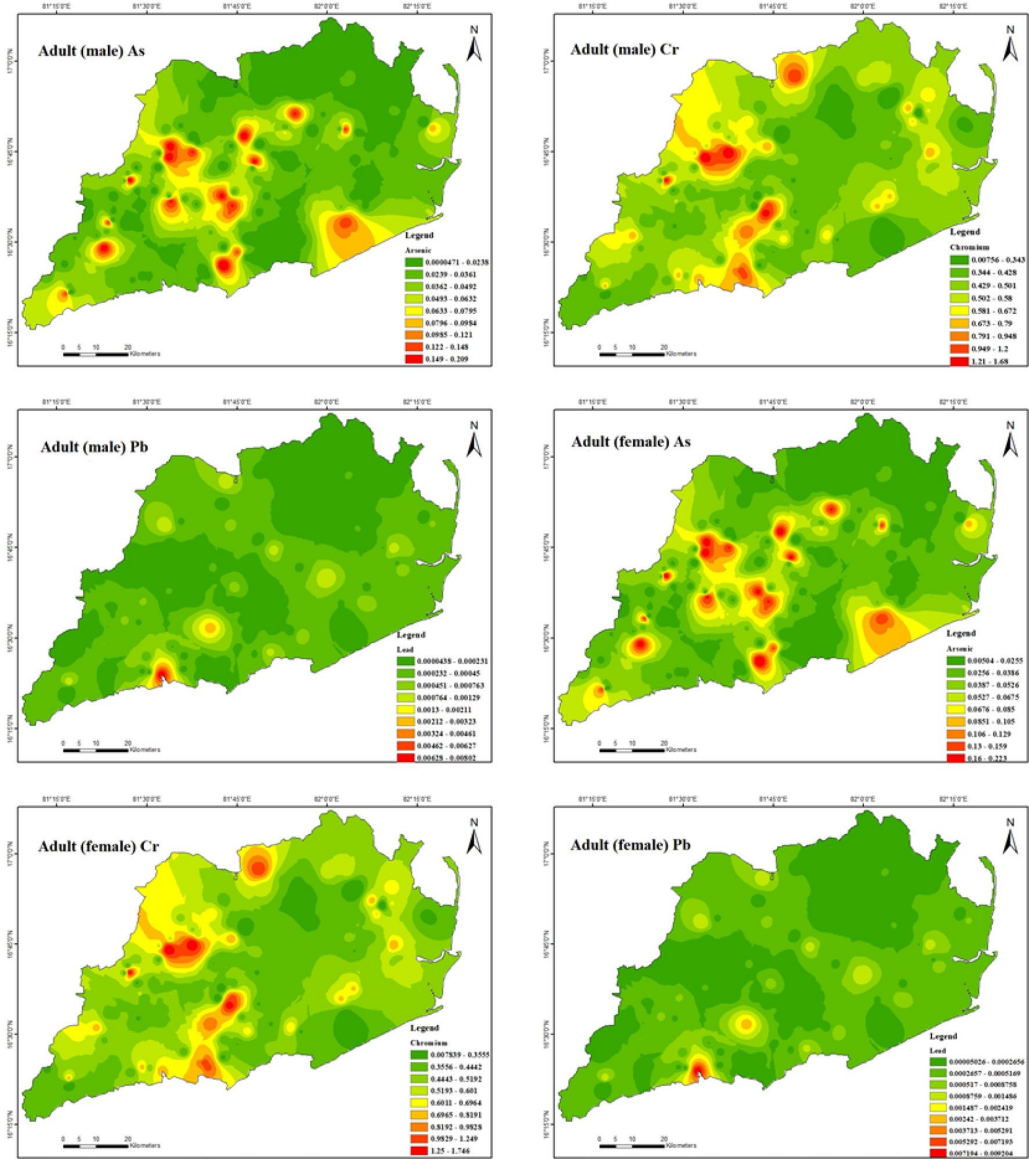
Spatial distribution of carcinogenic health risk due to As, Cr & Pb in Adult (male & female) (maps were generated with software ArcGIS 10.7 http://www.esri.com).

**Figure 13:**
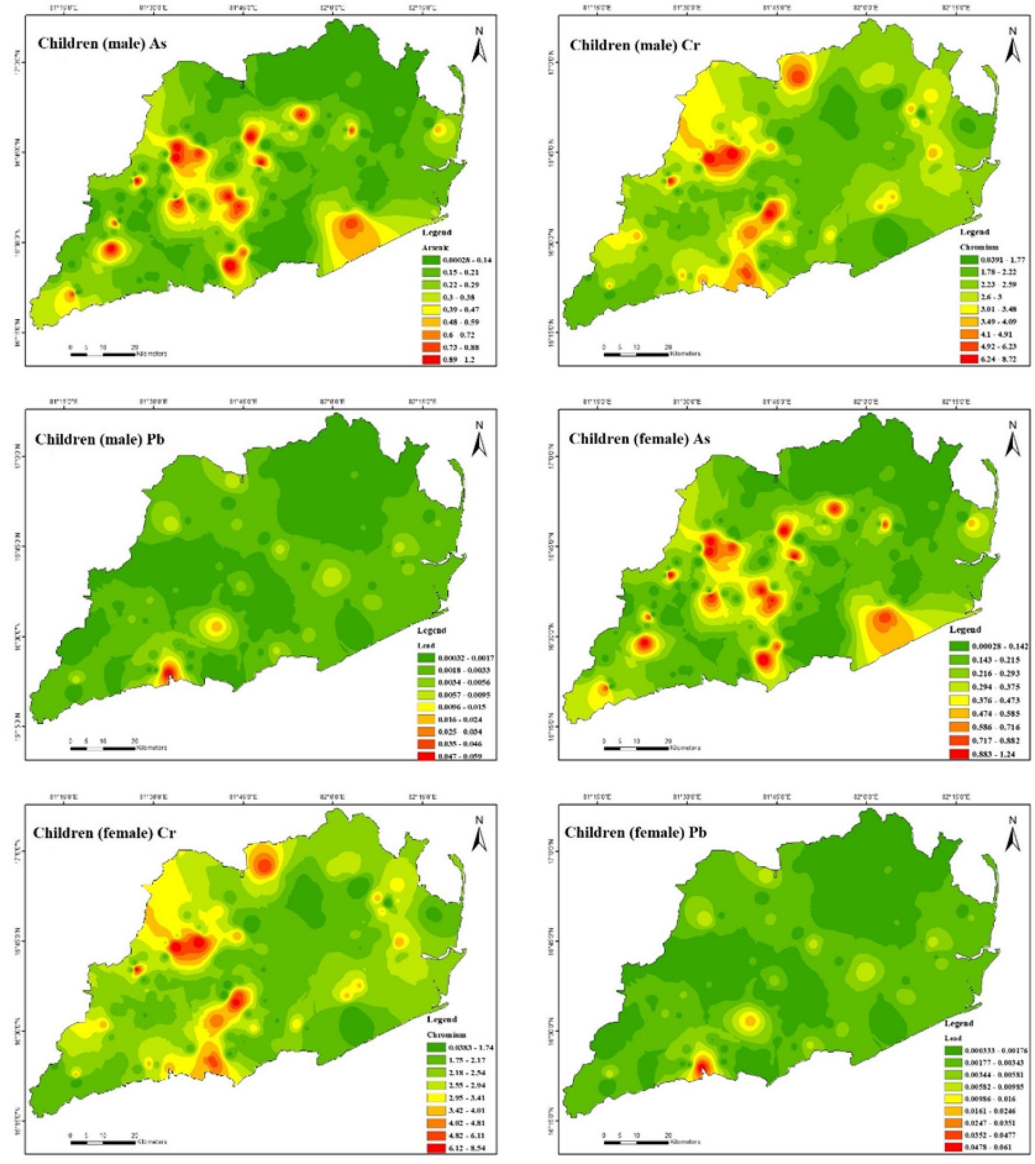
Spatial distribution of carcinogenic health risk due to As, Cr & Pb in Children (male& female) (maps were generated with software ArcGIS 10.7 http://www.esri.com).

Individuals are growing substantially the risk of cancer through being exposed to toxins including As, Cr, and Pb. The USEPA considers carcinogenic risks (CR) and total carcinogenic risks (TCR) levels below 1.0E − 06 to be minimal, whereas values exceeding 1.0E − 04 can become dangerous to human health (USEPA 1989, 2002). Table 11 depicts the results of 3 different treatment paths for As, Cr, and Pb for adults and children (male and female) (Figs. 12 and 13). However, the TCR of Cr for adults in both male and female ranged from 7.1E − 03 to 1.7E + 00 and 7.3E − 03 to 1.8E + 00 with a mean of 4.9E − 01 and 5.1E − 01 respectively; its acceptable threshold value of 1.0E − 04 was exceeded by about 97.8 and 98.5% of the sample locations, respectively (USEPA 2002). Further, TCR values of As and Cr for children in males and females varied from 1.1E − 04 to 1.2E + 00, 3.7E − 02 to 8.8E + 00, and 1.1E − 04 to 1.2E + 00, 3.6E − 02 to 8.6E + 00 with a mean of 2.5E − 01, 2.5E + 00 and 2.5E − 01, 2.5E + 00 respectively; About all of the sampled locations exceeded the threshold value, which is considered acceptable. The carcinogenic health hazards to adults and children from exposure to As and Cr have typically greater potential carcinogenic health risks when compared to other heavy metals, and the Pb risks posed to adults and children (male and female) from exposure were considerably less in the study area. Many authors have noticed that the surface soils surrounding an industrial place in India proved more carcinogenic to children than adults^104^. Many other areas have comparable results^68,105–109^. Few studies show that emerging soil pollution increased carcinogenic and non-carcinogenic health risks in every area^110^. It was also observed in a study that the levels of As and Cr in the soil of an example county in Shanxi Province, China exceeded the acceptable limit of 1.0E − 04^111^. This result suggests that children in the area may be exposed to carcinogenic risks through ingestion, dermal contact, and inhalation pathways. According to results, the heavy metals discharged from the drilling operations have significantly contaminated the soils close to the oil and gas drilling locations, according to health and environmental risk assessments. In addition, soil contamination with heavy metals remains a significant source of carcinogenic and non-carcinogenic hazards for the general population, with particular emphasis on vulnerable groups such as children and individuals residing in areas with the highest pollution levels.

**Table 11:** Results of carcinogenic risks through ingestion, inhalation, and dermal pathways for adults and children (male and female) in the study region.

	Carcinogenic	Adult male				Adult female			
		CR_ing_	CR_inh_	CR_der_	TCR	CR_ing_	CR_inh_	CR_der_	TCR
As	Minimum	5.8E − 06	7.6E − 12	1.3E − 05	1.9E − 05	6.7E − 06	7.5E − 12	1.4E − 05	2.0E − 05
	Maximum	0.06352	8.3E − 08	0.14586	2.1E − 01	0.07293	8.2E − 08	0.15087	2.2E − 01
	Mean	0.01279	1.7E − 08	0.02936	4.2E − 02	0.01468	1.7E − 08	0.03037	4.5E − 02
Cr	Minimum	0.00039	0.00064	0.00603	7.1E − 03	0.00045	0.00064	0.00623	7.3E − 03
	Maximum	0.09402	0.15473	1.4482	1.7E + 00	0.10794	0.15323	1.49795	1.8E + 00
	Mean	0.02704	0.0445	0.41655	4.9E − 01	0.03105	0.04407	0.43086	5.1E − 01
Pb	Minimum	4.4E − 05			4.4E − 05	5E − 05			5.0E − 05
	Maximum	0.00805			8.0E − 03	0.00924			9.2E − 03
	Mean	0.00033			3.3E − 04	0.00038			3.8E − 04

	Carcinogenic	Children male				Children female			
		CR_ing_	CR_inh_	CR_der_	TCR	CR_ing_	CR_inh_	CR_der_	TCR
As	Minimum	4.2E − 05	1.6E − 11	7.2E − 05	1.1E − 04	4.4E − 05	1.6E − 11	7E − 05	1.1E − 04
	Maximum	0.46387	1.8E − 07	0.78234	1.2E + 00	0.48354	1.8E − 07	0.76081	1.2E + 00
	Mean	0.09337	3.5E − 08	0.15747	2.5E − 01	0.09733	3.6E − 08	0.15314	2.5E − 01
Cr	Minimum	0.00286	0.00136	0.03233	3.7E − 02	0.00298	0.00138	0.03144	3.6E − 02
	Maximum	0.68658	0.32775	7.76753	8.8E + 00	0.71571	0.33266	7.55374	8.6E + 00
	Mean	0.19748	0.09427	2.23418	2.5E + 00	0.20586	0.09568	2.17342	2.5E + 00
Pb	Minimum	0.00032			3.2E − 04	0.00033			3.3E − 04
	Maximum	0.05876			5.9E − 02	0.06125			6.1E − 02
	Mean	0.00242			2.4E − 03	0.00253			2.5E − 03

### Potential health hazards

Various short- and long-term toxicological impacts of heavy metals have been observed to impact diverse internal organs. Heavy metals may cause gastrointestinal dysfunction, renal dysfunction, neurological diseases, skin lesions, vascular damage, immune system malfunction, birth abnormalities, and cancer. Cumulative effects may result from contemporaneous exposure to more than one metal^112–116^. Arsenic, considered a hazardous heavy metal, is a significant contributor to the endangerment of human health. The substance As has a lengthy historical record of use as a metalloid element and a therapeutic agent. The potential adverse outcomes encompass hyperpigmentation, cutaneous abnormalities, and elevated susceptibility to neoplastic growth. Cr has the potential to induce a range of ailments via its process of bioaccumulation within the human body. This includes skin, kidney, nervous system, and gastrointestinal diseases, as well as cancers of the lungs, throat, bladder, kidneys, ovaries, bones, and thyroid^117,118^. Pb may cause neurological, respiratory, urinary, and cardiovascular issues. Immune modulation, oxidative damage, and inflammation cause these consequences. Exposure to lead (Pb) was found to cause changes in the physiological functions of the human body and is linked to the development of numerous illnesses^119–121^.

## Conclusion

The study described the preliminary distribution of heavy metals in soils and their probable source distribution around oil and natural gas drilling sites and presented obvious disparities and varied correlations among selected metal contents (As, Cr, Cu, Ni, Pb and Zn) which were higher than their background values in the study area. According to the results, the higher concentration of heavy metals could be attributed to both natural and anthropogenic source of contamination, majority would be anthropogenic input from backwater waste generated during drilling. Apart from oil drilling sites, the other probable source of anthropogenic contamination due to heavy metals can be attributed as well due to agriculture which are active in the study area.

The multivariate revealed significant anthropogenic pollution especially by As, Cr, Cu and Pb in soils of study area. As a result of the index of geoaccumulation, enrichment factor and contamination factor very high As, Pb and Cr concentrations were found in the soil and may be mix with groundwater by leaching and posing serious threat to human health. These risk indices when applied to the soils of the study area revealed high risk of contamination by As, Pb and Cr and the order of ecological risk index was As > Pb > Ni > Cr > Cu > Zn. The risk of chronic disease occurrence is generally low in adults and medium to high in children. Some of the sampling points indicated high potential risk of As and Pb as these had medium to high chronic as well as carcinogenic risk. The study area shows a potentially greater risk due to anthropogenic contamination. Assessment of health risk from toxic elements like As, Pb and Cr contamination in soils have been mapped in the form of distribution maps which will highlight the potential risk zone.

## Recommendations

The recommendations in the study area would be to monitor the soil parameters by applying basic quality testing and to monitor the activities in the drilling sites. To further contain the backwater chemicals generated which are harmful to human health, biological treatment has been an effective but infrequently used for oil and gas produced water. Few biological treatments of produced water include information for fixed-film treatment, membrane bioreactors, wetlands and ponds, activated sludge treatment, anaerobic treatment, and bio-electrochemical treatment, and biopreparation for the bioremediation of oil-contaminated soils. Further, suggest suitable remedial measures to avert contamination due to heavy metals in near future.

## Data Availability

The datasets used and/or analysed during the current study available from the corresponding author on reasonable request.
